# Titanium-mediated reductive cross-coupling reactions of imines with terminal alkynes: An efficient route for the synthesis of stereodefined allylic amines

**DOI:** 10.3762/bjoc.9.69

**Published:** 2013-03-27

**Authors:** Kebin Mao, Guoqin Fan, Yuanhong Liu, Shi Li, Xu You, Dan Liu

**Affiliations:** 1 State Key Laboratory of Organometallic Chemistry, Shanghai Institute of Organic Chemistry, Chinese Academy of Sciences, 345 Lingling Lu, Shanghai 200032, People’s Republic of China; 2 College of Chemical Engineering, Shenyang University of Chemical Technology, Shenyang 110142, People’s Republic of China

**Keywords:** allylic amine, azatitanacyclopentene, reductive cross-coupling, regioselectivity, terminal alkyne, titanium-imine complex

## Abstract

Low-valency titanium species, generated in situ by using Ti(OiPr)_4_/2 *c*-C_5_H_9_MgCl reagent, react with imines to give a titanium-imine complex that can couple with terminal alkynes to provide azatitanacyclopentenes with excellent regioselectivity. Stereodefined allylic amines are obtained in good yields after hydrolysis or iodonolysis of the corresponding azatitanacyclopentenes. When ethynylcyclopropane is used as the coupling partner to react with imines in this reaction, the initially generated allylic amine undergoes an unexpected 1,3-amino migration on silica gel during the column chromatography.

## Introduction

Allylic amines are fundamental three-carbon building blocks in organic chemistry and their synthesis is an important industrial and synthetic goal [1–4]. The two functionalities in the allylic amine fragment, i.e., the nucleophilic amino group and the alkene, can ideally participate in cycloaddition reactions [5–6], condensation reactions [7], nucleophilic substitution reactions [8–9], radical reactions [10] and Pd-catalyzed reactions [11]. Thus, allylic amines have been used for the synthesis of numerous heterocycles and bioactive amines, such as α- and β-amino acids [12–15], different alkaloids [16], aminoallylsilanes [17], aminoepoxides [18], iodocyclocarbamates [19] and isoxazolines [20]. Although it has been reported that allylic amines can be synthesized by methods such as amination of allylic alcohols [21–24], direct allylic amination of simple alkenes [25–27], Morita–Baylis–Hillman reaction [28], alkenylation of imines [29–32], etc., it is still a great challenge to synthesize allylic amines with a stereodefined alkene moiety. The low-valency group 4 metal complexes (M = Ti or Zr) mediated reductive cross-coupling of imines with alkynes is one of the useful methods to construct stereodefined allylic amines. For example, Buchwald et al. reported that zirconocene-imine complexes, generated by treating Cp_2_ZrMeCl with lithium dialkylamide followed by elimination of methane from the resulting zirconocene(methyl) amide complex, coupled with alkynes to give geometrically pure allylic amines after hydrolysis [33]. They also developed an asymmetric variant of this reaction that proceeded to give allylic amine products with ee’s up to 99% by using chiral *ansa*-zirconocenes [34]. However, these reactions required a tedious multistep procedure for the preparation of zirconocene–imine complexes. In addition, the use of terminal alkynes produced an inseparable mixture of two regioisomers in some cases [33] or could not give the desired products [34]. Sato et al. reported that a divalent titanium reagent generated by the Ti(OiPr)_4_/2 iPrMgX system reacted with arylaldimines to provide the corresponding (η^2^-imine)Ti(OiPr)_2_ complex that, in turn, reacted with alkynes to give allylic amines after hydrolysis of the resulting azatitanacyclopentenes [35]. In this report, a terminal alkyne showed excellent regioselectivity and much better reactivity than internal alkynes. But only one successful example using a terminal alkyne appeared in this report (1-octyne). Sato’s group further applied this reaction for the synthesis of optically active allylic amines with chiral imines and terminal alkynes [36]. However, the imine substrates employed in their reactions were all *N*-alkyl substituted ones [35–36]. Until now the scope and limitations for titanium-mediated reductive cross-coupling reactions of imines with terminal alkynes have been far less studied.

**Scheme 1:**
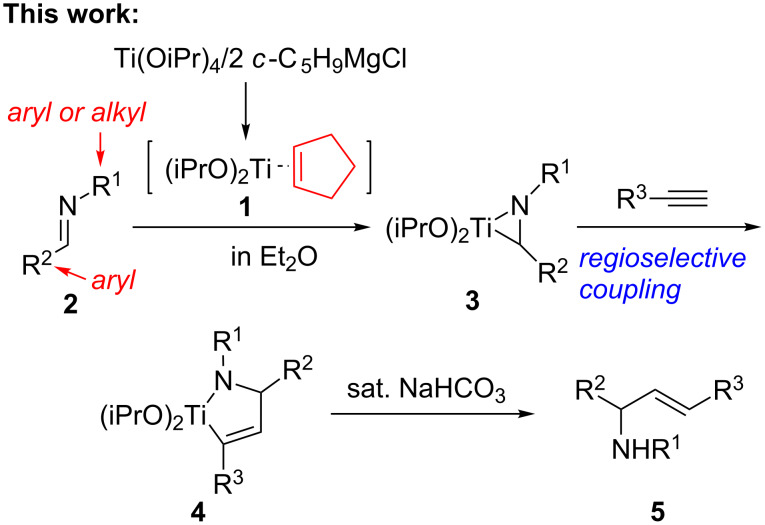
Titanium-mediated cross-coupling of imines with terminal alkynes.

Our group has developed a series of reactions using low-valency titanium reagents [37–39], including selective coupling of 1,3-butadiynes with aldehydes using Ti(OiPr)_4_/2 *n*-BuLi reagent [37] and titanium-mediated formation of *cis*-[3]cumulenes in the presence of a Lewis acid [38]. Very recently, we reported titanium mediated cross-coupling reactions of imines with ketones or aldehydes by the activation of imines with Ti(OiPr)_4_/2 *c*-C_5_H_9_MgCl reagent [40–42] leading to 1,2-amino alcohols [39]. These results prompted us to study the cross-coupling of imines with terminal alkynes by using the Ti(OiPr)_4_/2 *c*-C_5_H_9_MgCl reagent. In this paper, we describe the detailed results of these reactions (Scheme 1).

## Results and Discussion

**Scheme 2:**
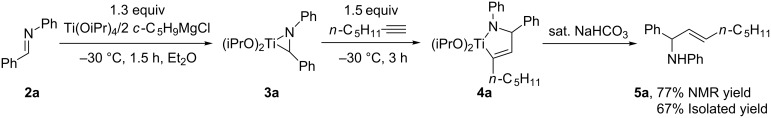
Synthesis of allylic amine **5a** by titanium-mediated coupling reaction of imine **2a** with 1-heptyne.

**Scheme 3:**
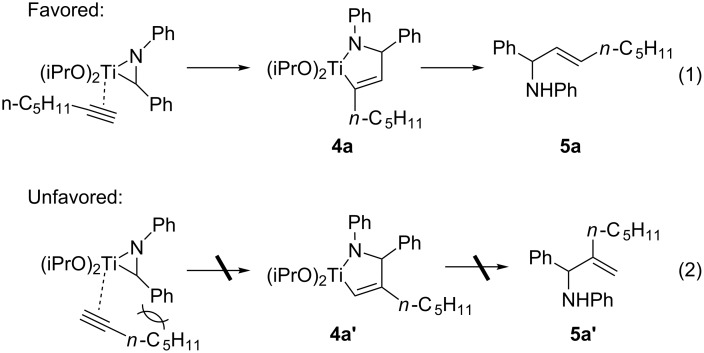
The regiochemistry of titanium-mediated cross-coupling of imine with terminal alkyne.

**Synthesis of allylic amines by reductive cross-coupling using Ti(OiPr)_4_/2 *c*-C_5_H_9_MgCl.** First, a typical example for the synthesis of allylic amines by reductive cross-coupling reactions using Ti(OiPr)_4_/2 *c*-C_5_H_9_MgCl reagent was studied by using imine **2a** and 1-heptyne as model substrates (Scheme 2). Based on our previous report [39], Ti-imine complex **3a** was generated in situ by the reaction of imine **2a** with 1.3 equiv of Ti(OiPr)_4_/2 *c*-C_5_H_9_MgCl at −30 °C. It was found that Ti-imine complex **3a** could smoothly couple with 1.5 equiv of 1-heptyne to give allylic amine **5a** in 77% NMR yield after hydrolysis of the resulting azatitanacyclopentene complex **4a** with saturated aqueous NaHCO_3_ solution. In this reaction, azatitanacyclopentene **4a**, rather than its regioisomer **4a’**, was formed preferentially, in which the pentyl group is situated adjacent to titanium (Scheme 3, reaction 1). Accordingly, the allylic amine **5a** could be obtained after hydrolysis with excellent regioselectivity. There was no apparent formation of the regioisomer **4a’** and allylic amine **5a’** in this reaction, which may be due to the strong steric repulsion between the phenyl and pentyl groups during the coupling process (Scheme 3, reaction 2).

**Figure 1:**
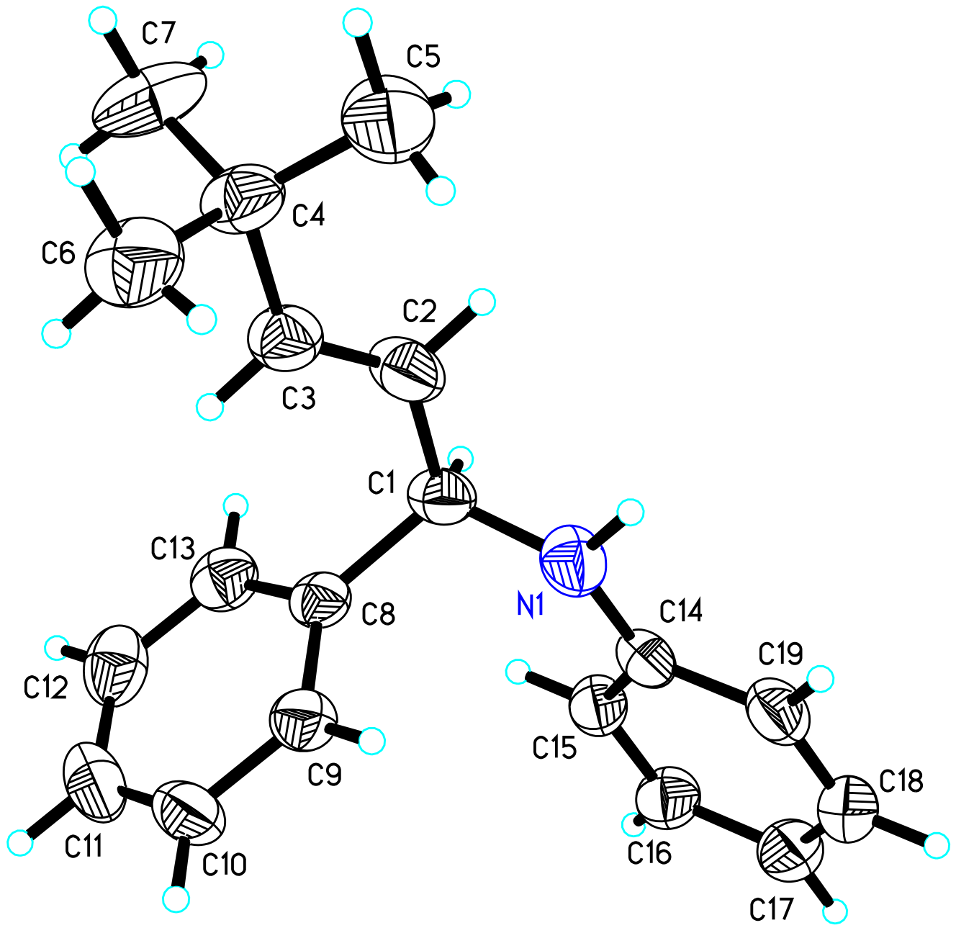
X-ray crystal structure of compound **5c**.

**Reaction scope of various terminal alkynes and imines.** With the optimized reaction conditions in hand, we next investigated the reaction scope by first performing the reaction of imine **2a** with various terminal alkynes as shown in Table 1. When the terminal alkynes with *n*-hexyl or *tert*-butyl groups were used as coupling partners to react with imine **2a**, the corresponding allylic amines **5b–c** were obtained in 69–88% yields (Table 1, entries 2 and 3). The (*E*)-configuration of allylic amines **5** was confirmed by X-ray crystal analysis of **5c** as shown in Figure 1 [43]. Terminal alkynes with chloro- or phenyl-functionalized alkyl chains were both compatible with this coupling reaction, furnishing the corresponding products **5d** and **5e** in 66% and 68% yields, respectively (Table 1, entries 4 and 5). Even terminal alkynes with trimethylsilyl or 2-pyridyl functionalities were tolerated well during the reaction to give allylic amines **5f** and **5g** in 80% and 81% yields, respectively (Table 1, entries 6 and 7).

**Table 1:** Synthesis of various allylic amines by titanium-mediated coupling reactions of imine **2a** with different terminal alkynes.

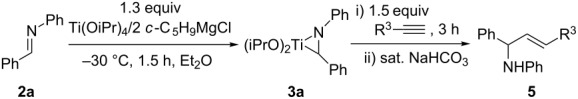			

entry	terminal alkyne	product	yield (%) of **2a**^a^

1	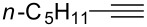	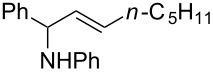 **5a**	67
2	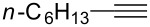	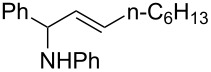 **5b**	69
3	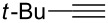	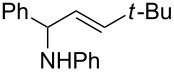 **5c**	88
4	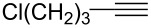	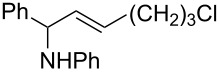 **5d**	66
5	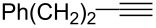	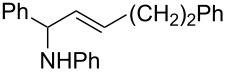 **5e**	68
6	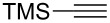	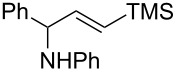 **5f**	80
7	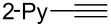	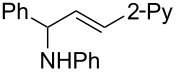 **5g**	81

^a^Isolated yields.

A broad range of imine substrates were also examined for this reaction, as shown in Table 2. When the cross-coupling reactions of *N*-(*p*-bromophenyl)- or *N*-(*p*-methoxyphenyl)-substituted imines **2b** and **2c** were employed with 2-ethynylpyridine under the same conditions, the corresponding allylic amines **5h** and **5i** were obtained in 84% and 80% yields, respectively (Table 2, entries 1 and 2). The results indicated that electron-poor or -rich aryl substituents on the nitrogen atom of imines **2** had little influence on the yields of products **5**. The reaction of imine **2d**, with a bulky *N*-(1-naphthyl) group, with *t*-Bu-substituted alkyne also proceeded well to give allylic amine **5j** in 67% yield (Table 2, entry 3). *C*-(*p*-bromophenyl)- or *C*-(*p*-methoxyphenyl)-substituted imines **2e** and **2f** reacted well with a series of terminal alkynes, furnishing **5k**–**5n** in 75–84% yields (Table 2, entries 4–7). The results indicated that the electronic nature of the *C*-aryl ring also had little influence on the product yields. The reaction of *N*-propyl-substituted imine **2g** with 2-ethynylpyridine produced the corresponding allylic amine **5o** in 60% yield (Table 2, entry 8). In contrast to the results obtained by using Ti(OiPr)_4_/2 iPrMgX reagent [35], the coupling of imine **2g** with 1-octyne could not afford the desired coupling product in our system (Table 2, entry 9). The structure of allylic amines was also determined by X-ray crystal analyses of compounds **5h** and the acylated derivative (**7**) of **5l** [43].

**Table 2:** Synthesis of various allylic amines by titanium-mediated coupling reactions of different imines with terminal alkynes.

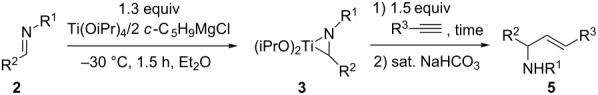					

entry	imine	terminal alkyne	time (h)^a^	product	yield (%)^b^

1	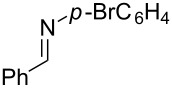 **2b**	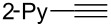	6	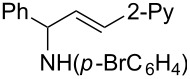 **5h**	84
2	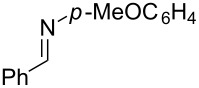 **2c**	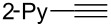	6	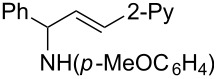 **5i**	80
3^c^	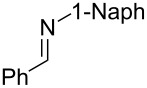 **2d**	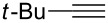	4	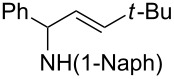 **5j**	67
4	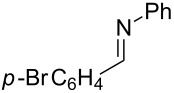 **2e**	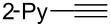	5	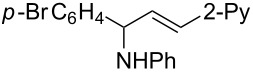 **5k**	81
5	**2e**	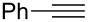	6	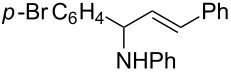 **5l**	81
6	**2e**	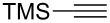	3	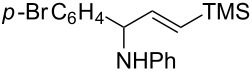 **5m**	84
7	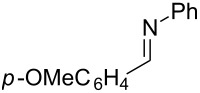 **2f**	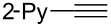	6	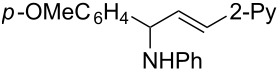 **5n**	75
8	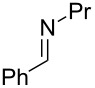 **2g**	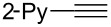	6	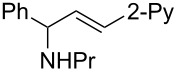 **5o**	60
9	**2g**	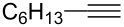	3	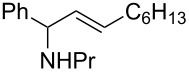 **5p**	–^d^

^a^Reaction time for the second step. ^b^Isolated yields. ^c^1-Naph is 1-naphthyl. ^d^The desired product was not isolated.

**Scheme 4:**
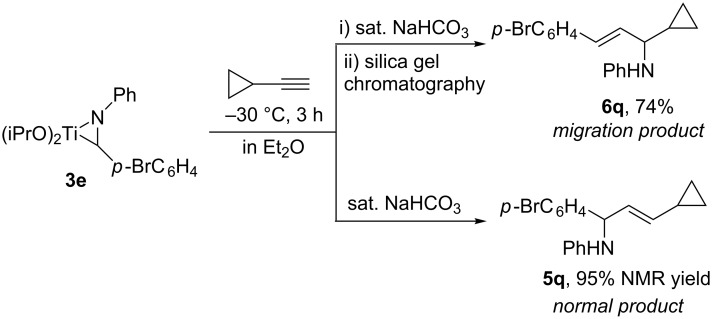
Synthesis of allylic amines **5q** and **6q**.

**Figure 2:**
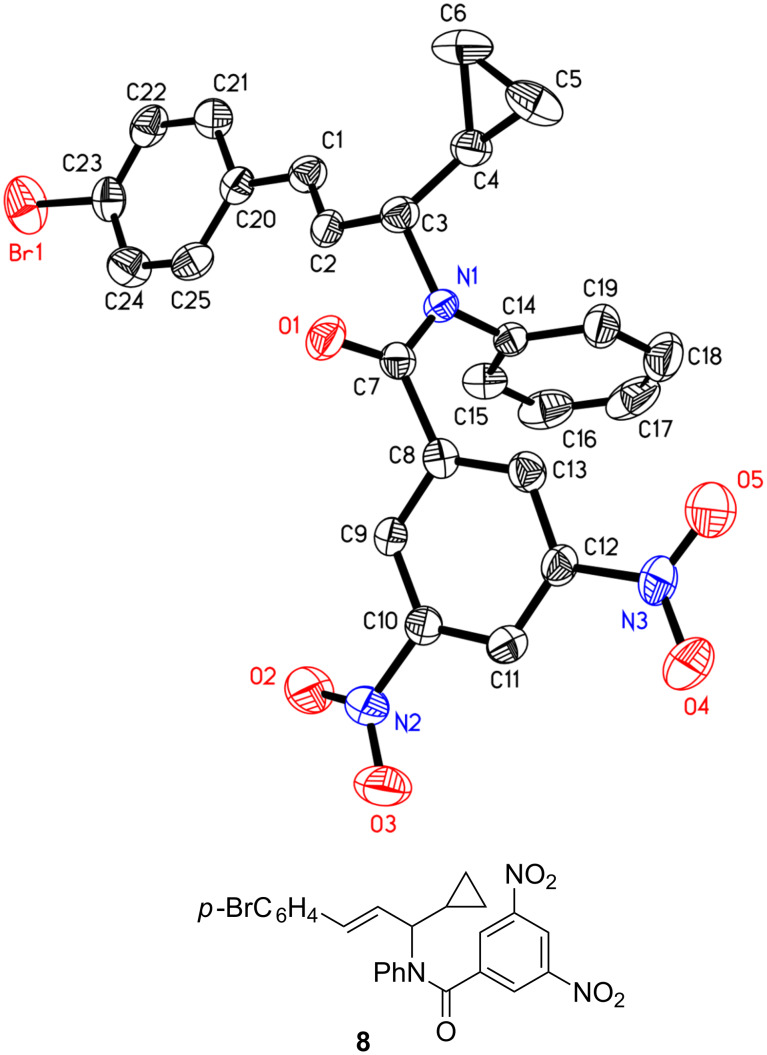
X-ray crystal structure of compound **8**.

**Titanium-mediated reductive cross-coupling reaction of imines with ethynylcyclopropane.** When ethynylcyclopropane was used as the coupling partner of imines **2** in the titanium-mediated reaction, 1,3-amino group migration occurred unexpectedly during the purification of the products by silica-gel chromatography (Scheme 4). For example, the reaction of azatitanacyclopropene **3e** with 1.5 equiv of ethynylcyclopropane at −30 °C for 3 h afforded, after silica-gel chromatography, the amino-migration product of 1-cyclopropyl allylic amine **6q** in 74% yield. The structure of **6q** was confirmed unambiguously by X-ray crystal analysis of its amide derivative **8** ((*E*)-*N*-(3-(4-bromophenyl)-1-cyclopropylallyl)-3,5-dinitro-*N*-phenylbenzamide) as shown in Figure 2 [43]. Careful analysis of the crude reaction mixture before silica-gel purification revealed that the normal coupling product **5q** was observed in 94% NMR yield. The result indicated that an isomerization of **5q** to **6q** occurred during the silica-gel isolation process. This isomerization may proceed via the formation of an allyl cationic intermediate promoted by silica gel due to its weak Lewis acidity [44–45].

**Scheme 5:**
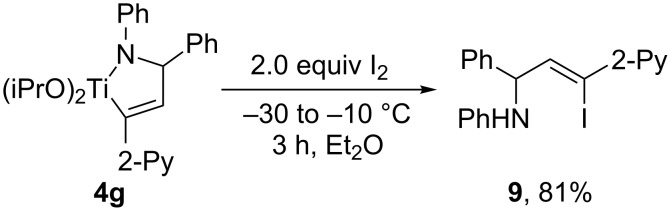
Synthesis of allylic amine **9** by iodonolysis of azatitanacyclopentene **4g**.

**Iodonolysis of azatitanacyclopentene.** Furthermore, we found that iodinated allylic amine **9** could be obtained by iodonolysis of the azatitanacyclopentenes **4** (Scheme 5). For example, on treatment of azatitanacyclopentene **4g** with two equiv of iodine at −30 °C followed by warming to −10 °C and stirring for 3 h, iodinated allylic amine **9** could be isolated in 81% yield. Compound **9** is highly valuable since further functionalization could be explored to synthesize a wide range of organic molecules.

## Conclusion

In conclusion, we have developed efficient reductive cross-coupling reactions of imines with terminal alkynes by the activation of imines using Ti(OiPr)_4_/2 *c*-C_5_H_9_MgCl reagent. Various substituted allylic amine derivatives were obtained in good yields and with excellent regioselectivity after hydrolysis or iodonolysis of the resulting azatitanacyclopentenes. Further studies on the synthetic utility of the resulting titanacyclic intermediates and allylic amines are currently in progress.

## Acknowledegments

We thank the National Natural Science Foundation of China (Grant No. 21072208, 21125210, 20821002), Chinese Academy of Science, and the Major State Basic Research Development Program (Grant No. 2011CB808700) for financial support.

## Supporting Information

File 1 Experimental section and NMR spectra.

## References

[1] Lawrence S A (2004). Amines: Synthesis, Properties and Applications.

[2] Cheikh R B, Chaabouni R, Laurent A, Mison P, Nafti A (1983). Synthesis.

[3] Johannsen M, Jørgensen K A (1998). Chem Rev.

[4] Nag S, Batra S (2011). Tetrahedron.

[5] Sunderhaus J D, Dockendorff C, Martin S F (2009). Tetrahedron.

[6] Pöverlein C, Breckle G, Lindel T (2006). Org Lett.

[7] Timoshchuk V A, Hogrefe R I (2009). Nucleosides, Nucleotides Nucleic Acids.

[8] Warmus J S, Dilley G J, Meyers A I (1993). J Org Chem.

[9] Monbaliu J-C, Marchand-Brynaert J (2008). Tetrahedron Lett.

[10] Lee H S, Kim H S, Kim J M, Kim J N (2008). Tetrahedron.

[11] Scarborough C C, Stahl S S (2006). Org Lett.

[12] Hayashi T, Yamamoto A, Ito Y, Nishioka E, Miura H, Yanagi K (1989). J Am Chem Soc.

[13] Jumnah R, Williams J M J, Williams A C (1993). Tetrahedron Lett.

[14] Burgess K, Liu L T, Pal B (1993). J Org Chem.

[15] Bower J F, Jumnah R, Williams A C, Williams J M J (1997). J Chem Soc, Perkin Trans 1.

[16] Magnus P, Lacour J, Coldham I, Mugrage B, Bauta W B (1995). Tetrahedron.

[17] Franciotti M, Mordini A, Taddei M (1992). Synlett.

[18] Luly J R, Dellaria J F, Plattner J J, Soderquist J L, Yi N (1987). J Org Chem.

[19] Kobayashi S, Isobe T, Ohno M (1984). Tetrahedron Lett.

[20] Nishi T, Morisawa Y (1989). Heterocycles.

[21] Sen S E, Roach S L (1995). Synthesis.

[22] Overman L E, Zipp G G (1997). J Org Chem.

[23] Guo S, Song F, Liu Y (2007). Synlett.

[24] Defieber C, Ariger M A, Moriel P, Carreira E M (2007). Angew Chem, Int Ed.

[25] Katz T J, Shi S (1994). J Org Chem.

[26] Brucko M, Khuong T-A V, Sharpless K B (1996). Angew Chem, Int Ed Engl.

[27] Shimizu Y, Obora Y, Ishii Y (2010). Org Lett.

[28] Rastogi N, Mohan R, Panda D, Mobin S M, Namboothiri I N N (2006). Org Biomol Chem.

[29] Oi S, Moro M, Fukuhara H, Kawanishi T, Inoue Y (1999). Tetrahedron Lett.

[30] Wipf P, Kendall C, Stephenson C R J (2003). J Am Chem Soc.

[31] Brak K, Ellman J A (2009). J Am Chem Soc.

[32] Li Y, Xu M-H (2012). Org Lett.

[33] Buchwald S L, Watson B T, Wannamaker M W, Dewan J C (1989). J Am Chem Soc.

[34] Grossman R B, Davis W M, Buchwald S L (1991). J Am Chem Soc.

[35] Gao Y, Yoshida Y, Sato F (1997). Synlett.

[36] Fukuhara K, Okamoto S, Sato F (2003). Org Lett.

[37] Chen J, Liu Y (2008). Tetrahedron Lett.

[38] Chen J, Liu Y (2010). Organometallics.

[39] Fan G, Liu Y (2012). Tetrahedron Lett.

[40] Lecornué F, Ollivier J (2003). Chem Commun.

[41] McLaughlin M, Takahashi M, Micalizio G C (2007). Angew Chem, Int Ed.

[42] Chen M Z, Micalizio G C (2009). Org Lett.

[R43] 43CCDC-910581 (**5c**), 910580 (**5h**), 910575 (**7**), 910582 (**8**) contain the supplementary crystallographic data for this paper. These data can be obtained free of charge from The Cambridge Crystallographic Data Centre via http://www.ccdc.cam.ac.uk/data_request/cif.

[44] Shao L-X, Li Y-X, Shi M (2007). Chem–Eur J.

[45] Eşsiz S, Şengül M E, Şahin E, Daştan A (2011). Turk J Chem.

