# Supplementary material for: Titanium-mediated reductive cross-coupling reactions of imines with terminal alkynes: An efficient route for the synthesis of stereodefined allylic amines
Source: Beilstein J Org Chem. 2013 Mar 27;9:621–7. doi: 10.3762/bjoc.9.69 (PMC3628913; doi:10.3762/bjoc.9.69)

Supporting Information

for

Titanium-mediated reductive cross-coupling reactions of imines with terminal alkynes: An efficient route for the synthesis of stereodefined allylic amines

Kebin Mao[1], Guoqin Fan[2], Yuanhong Liu[2,*,§], Shi Li[2], Xu You[2] and Dan Liu[1]

Address: [1]College of Chemical Engineering, Shenyang University of Chemical Technology, Shenyang 110142, People's Republic of China and [2]State Key Laboratory of Organometallic Chemistry, Shanghai Institute of Organic Chemistry, Chinese Academy of Sciences, 345 Lingling Lu, Shanghai 200032, People's Republic of China

Email: Yuanhong Liu - *yhliu@mail.sioc.ac.cn*

*Corresponding author

[§]Tel:+86-21-54925135; Fax: +86-21-64166128

Experimental section and NMR spectra

General Methods

All reactions were carried out using standard Schlenk techniques under argon. Diethyl ether was distilled from sodium and benzophenone. Titanium(IV) isopropoxide was purchased from TCI Chemical Company. Cyclopentylmagnesium chloride (2.0 M solution in diethyl ether) was purchased from Aldrich Co. Ltd. and was used as soon as possible after opening. Unless noted, all commercial reagents were used without further purification. Imines were prepared according to standard procedures and the spectroscopic data of these imines are in agreement with those previously reported.

^{1}H and ^{13}C NMR spectra were recorded at room temperature in $CDCl_3$ (containing 0.03% tetramethylsilane) on Varian XL-300 MHz spectrometer or Varian XL-400 MHz spectrometer. ^{1}H NMR spectra were recorded at 300 or 400 MHz, ^{13}C NMR spectra were recorded at 75.5 or 100.6 MHz. ^{1}H NMR spectra was recorded with tetramethylsilane (δ = 0.00 ppm) as internal reference; ^{13}C NMR spectra was recorded with $CDCl_3$ (δ = 77.00 ppm) as internal reference. High-resolution mass spectra were obtained by using a Waters Micromass GCT Premier or an Agilent Technologies 6224 TOF LC/MS mass spectrometer. Elemental analyses were performed on an Italian Carlo-Erba 1106 analyzer. Single-crystal X-ray diffraction data was collected on a Bruker SMART diffractometer. In some cases, purification was performed on recycling preparative HPLC (LC-92XX NEXT series).

Typical procedure for the cross-coupling reaction of imines with 1-alkynes.

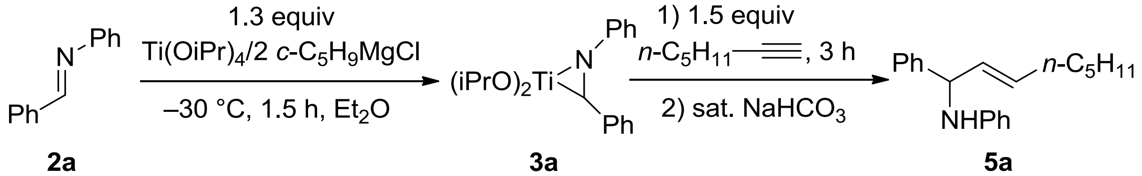

2a **3a** **5a**

To a stirred solution of imine **2a** (91 mg, 0.5 mmol) and $Ti(OiPr)_4$ (0.19 mL, 0.65 mmol) in Et_2O (5 mL) was added dropwise c-C_5H_9MgCl (0.65 mL, 2.0 M solution in diethyl ether, 1.3 mmol) at −30 °C. The solution was stirred at this temperature for 1.5 h, and then 1-heptyne (99 μL, 72 mg, 0.75 mmol) was added to the reaction mixture at −30 °C. After stirring for 3 h at −30 °C, the mixture was quenched by saturated aqueous $NaHCO_3$ solution and stirred for ca. 1–2 h at room temperature. The resulting mixture was extracted

with diethyl ether three times. The combined extract was washed separately with water and brine, and dried over anhydrous Na_2SO_4. The solvent was evaporated in vacuo, and the residue was purified by preparative TLC on silica gel (eluent: petroleum ether/ethyl acetate = 100/1) to afford the desired allylic amine **5a** as a yellow oil (94 mg, 67% isolated yield).

(*E*)-*N*-(1-Phenyloct-2-enyl)benzenamine (5a). ^{1}H NMR (300 MHz, $CDCl_3$, Me_4Si) δ 0.87 (t, *J* = 7.2 Hz, 3H), 1.22–1.43 (m, 6H), 1.99–2.06 (m, 2H), 4.02 (bs, 1H), 4.86 (d, *J* = 5.1 Hz, 1H), 5.57–5.72 (m, 2H), 6.56 (d, *J* = 8.4 Hz, 2H), 6.67 (t, *J* = 7.2 Hz, 1H), 7.11 (t, *J* = 7.8 Hz, 2H), 7.22–7.38 (m, 5H); ^{13}C NMR (75.5 MHz, $CDCl_3$, Me_4Si) δ 14.05, 22.46, 28.78, 31.35, 32.21, 60.42, 113.49, 117.35, 126.94, 127.10, 128.59, 129.02, 131.05, 132.90, 142.77, 147.31; HRMS–EI: calcd for $C_{20}H_{25}N$: 279.1987, found: 279.1986.

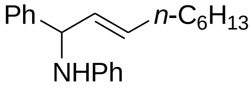

5b

(*E*)-*N*-(1-Phenylnon-2-enyl)benzenamine (5b). Purification of the crude product by preparative TLC on silica gel (eluent: petroleum ether/ethyl acetate = 100/1) afforded the title compound as a colorless oil in 69% yield. ^{1}H NMR (300 MHz, $CDCl_3$, Me_4Si) δ 0.87 (t, *J* = 6.9 Hz, 3H), 1.25–1.50 (m, 8H), 1.99–2.06 (m, 2H), 4.01 (bs, 1H), 4.86 (d, *J* = 5.1 Hz, 1H), 5.56–5.72 (m, 2H), 6.56 (d, *J* = 7.5 Hz, 2H), 6.66 (t, *J* = 7.5 Hz, 1H), 7.08–7.13 (m, 2H), 7.20–7.38 (m, 5H); ^{13}C NMR (75.5 MHz, $CDCl_3$, Me_4Si) δ 14.07, 22.60, 28.81, 29.06, 31.64, 32.26, 60.42, 113.49, 117.35, 126.94, 127.11, 128.60, 129.03, 131.06, 132.90, 142.77, 147.32; HRMS–EI: calcd for $C_{21}H_{27}N$: 293.2144, found: 293.2142.

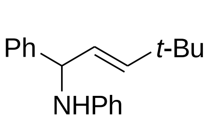

5c

***(E)*-*N*-(4,4-Dimethyl-1-phenylpent-2-enyl)benzenamine (5c).** Purification of the crude product by flash chromatography on silica gel (eluent: petroleum ether/ethyl acetate = 100/1) afforded the title compound as a white solid in 88% yield. ^{1}H NMR (300 MHz, $CDCl_3$, Me_4Si) δ 1.00 (s, 9H), 3.99 (bs, 1H), 4.85 (d, *J* = 6.6 Hz, 1H), 5.50 (dd, *J* = 6.6, 15.6 Hz, 1H), 5.72 (d, *J* = 15.6 Hz, 1H), 6.56 (d, *J* = 8.4 Hz, 2H), 6.66 (t, *J* = 7.5 Hz, 1H), 7.08–7.13 (m, 2H), 7.19–7.37 (m, 5H); ^{13}C NMR (75.5 MHz, $CDCl_3$, Me_4Si) δ 29.44,

32.95, 60.57, 113.52, 117.33, 125.94, 126.94, 127.05, 128.57, 128.99, 142.86, 143.59, 147.36; HRMS–EI: calcd for $C_{19}H_{23}N$: 265.1830, found: 265.1826.

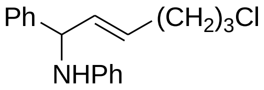

5d

***(E)*-*N*-(6-Chloro-1-phenylhex-2-enyl)benzenamine (5d).** Purification of the crude product by preparative TLC on silica gel (eluent: petroleum ether/ethyl acetate = 100/1) afforded the title compound as a yellow oil in 66% yield. ^{1}H NMR (400 MHz, $CDCl_3$, Me_4Si) δ 1.79–1.86 (m, 2H), 2.20 (dt, *J* = 6.4, 7.2 Hz, 2H), 3.47 (td, *J* = 6.4, 2.0 Hz, 2H), 4.01 (bs, 1H), 4.88 (d, *J* = 5.6 Hz, 1H), 5.60–5.73 (m, 2H), 6.56 (d, *J* = 7.2 Hz, 2H), 6.67 (t, *J* = 7.2 Hz, 1H), 7.09–7.13 (m, 2H), 7.22–7.27 (m, 1H), 7.31–7.37 (m, 4H); ^{13}C NMR (100 MHz, $CDCl_3$, Me_4Si) δ 29.25, 31.73, 44.25, 60.17, 113.48, 117.48, 126.94, 127.26, 128.65, 129.02, 130.44, 132.49, 142.34, 147.14; HRMS–EI: calcd for $C_{18}H_{20}NCl$: 285.1284, found: 285.1286.

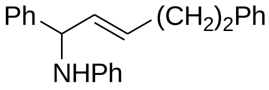

5e

(*E*)-*N*-(1,5-Diphenylpent-2-enyl)benzenamine (5e). Purification of the crude product by preparative TLC on silica gel (eluent: petroleum ether/ethyl acetate = 20/1) afforded the title compound as colorless oil in 68% yield. ^{1}H NMR (300 MHz, $CDCl_3$, Me_4Si) δ 2.35 (td, *J* = 6.6, 6.9 Hz, 2H), 2.67 (t, *J* = 7.2 Hz, 2H), 3.93 (bs, 1H), 4.83 (d, *J* = 6.0 Hz, 1H), 5.55–5.73 (m, 2H), 6.54 (d, *J* = 7.8 Hz, 2H), 6.70 (t, *J* = 7.2 Hz, 1H), 7.08–7.34 (m, 12H); ^{13}C NMR (75.5 MHz, $CDCl_3$, Me_4Si) δ 34.02, 35.47, 60.20, 113.44, 117.37, 125.80, 126.97, 127.15, 128.24, 128.54, 128.59, 129.03, 131.57, 131.89, 141.55, 142.48, 147.25; HRMS–EI: calcd for $C_{23}H_{23}N$: 313.1830, found: 313.1833.

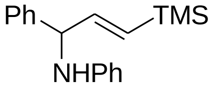

5f

***(E)*-*N*-(1-Phenyl-3-(trimethylsilyl)allyl)benzenamine (5f).** Purification of the crude product by preparative TLC on silica gel (eluent: petroleum ether/ethyl acetate = 100/1) afforded the title compound as a white solid in 80% yield. ^{1}H NMR (300 MHz, $CDCl_3$,

Me_4Si) δ 0.20 (s, 9H), 4.20 (bs, 1H), 5.05 (d, *J* = 5.4 Hz, 1H), 6.07 (d, *J* = 18.6 Hz, 1H), 6.32 (dd, *J* = 5.1, 18.6 Hz, 1H), 6.71 (d, *J* = 8.4 Hz, 2H), 6.81 (t, *J* = 7.2 Hz, 1H), 7.26 (t, *J* = 7.5 Hz, 2H), 7.33–7.49 (m, 5H); ^{13}C NMR (75.5 MHz, $CDCl_3$, Me_4Si) δ −1.31, 62.67, 113.47, 117.43, 127.20, 127.29, 128.68, 129.03, 130.84, 141.97, 145.93, 147.25; HRMS–EI: calcd for $C_{18}H_{23}NSi$: 281.1600, found: 281.1595.

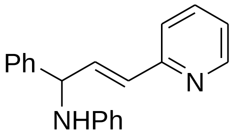

5g

***(E)*-*N*-(1-Phenyl-3-(pyridin-2-yl)allyl)benzenamine (5g).** Purification of the crude product by flash chromatography on silica gel (eluent: petroleum ether/ethyl acetate = 20/1) afforded the title compound as a dark red oil in 81% yield. ^1H NMR (400 MHz, $CDCl_3$, Me_4Si) δ 4.20 (bs, 1H), 5.13 (d, *J* = 6.0 Hz, 1Hz), 6.61–6.63 (m, 2H), 6.66–6.71 (m, 2H), 6.93 (dd, *J* = 6.4, 15.6 Hz, 1H), 7.06–7.15 (m, 3H), 7.19–7.21 (m, 1H), 7.24–7.28 (m, 1H), 7.32–7.35 (m, 2H), 7.41–7.43 (m, 2H), 7.53–7.58 (m, 1H), 8.51–8.52 (m, 1H); ^{13}C NMR (100 MHz, $CDCl_3$, Me_4Si) δ 60.21, 113.48, 117.64, 121.87, 122.16, 127.20, 127.54, 128.79, 129.07, 130.53, 135.11, 136.40, 141.51, 147.05, 149.43, 154.98; HRMS–EI: calcd for $C_{20}H_{18}N_2$: 286.1470, found: 286.1472.

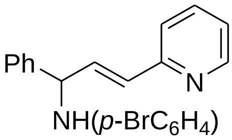

5h

***(E)*-4-Bromo-*N*-(1-phenyl-3-(pyridin-2-yl)allyl)benzenamine (5h).** Purification of the crude product by flash chromatography on silica gel (eluent: petroleum ether/ethyl acetate = 10/1) afforded the title compound as a red oil in 84% yield, which can be solidified at lower temperature. ^1H NMR (300 MHz, $CDCl_3$, Me_4Si) δ 4.26 (bs, 1H), 5.08 (d, *J* = 5.4 Hz, 1H), 6.49 (d, *J* = 8.7 Hz, 2H), 6.66 (d, *J* = 15.3 Hz, 1H), 6.91 (dd, *J* = 5.4, 15.3 Hz, 1H), 7.09–7.13 (m, 1H), 7.18–7.41 (m, 8H), 7.56–7.61 (m, 1H), 8.52 (d, *J* = 4.8 Hz, 1H); ^{13}C NMR (75.5 MHz, $CDCl_3$, Me_4Si) δ 60.15, 109.29, 115.07, 121.97, 122.32, 127.17, 127.74, 128.88, 130.72, 131.76, 134.51, 136.51, 140.96, 145.94, 149.46, 154.76; HRMS–ESI: calcd for $C_{20}H_{18}BrN_2$ $[M + H]^+$: 365.0653, found: 365.0638.

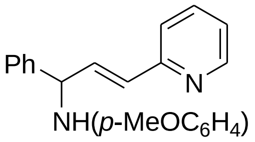

5i

***(E)*-4-Methoxy-*N*-(1-phenyl-3-(pyridin-2-yl)allyl)benzenamine (5i).** Purification of the crude product by flash chromatography on silica gel (eluent: petroleum ether/ethyl acetate = 10/1) afforded the title compound as a red oil in 80% yield. ^{1}H NMR (300 MHz, $CDCl_3$, Me_4Si) δ 3.69 (s, 3H), 5.05 (d, *J* = 6.0 Hz, 1H), 6.57–6.74 (m, 5H), 6.92 (dd, *J* = 6.0, 15.9 Hz, 1H), 7.08–7.09 (m, 1H), 7.20–7.44 (m, 6H), 7.53–7.59 (m, 1H), 8.51 (d, *J* = 4.5 Hz, 1H); The N–H proton was not detected; ^{13}C NMR (75.5 MHz, $CDCl_3$, Me_4Si) δ 55.60, 61.06, 114.63, 114.75, 121.81, 122.12, 127.16, 127.46, 128.74, 130.34, 135.45, 136.40, 141.26, 141.72, 149.39, 152.08, 155.00; HRMS–EI: calcd for $C_{21}H_{20}N_2O$: 316.1576, found: 316.1580.

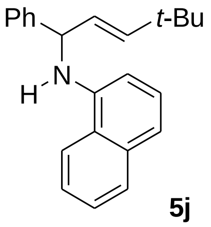

5j

(*E*)-*N*-(4,4-Dimethyl-1-phenylpent-2-enyl)naphthalen-1-amine (5j). Purification of the crude product by preparative TLC on silica gel (eluent: petroleum ether/ethyl acetate = 100/1) afforded the title compound as a white solid in 67% yield. ^{1}H NMR (400 MHz, $CDCl_3$, Me_4Si) δ 1.02 (s, 9H), 4.68 (bs, 1H), 5.03 (d, *J* = 6.4 Hz, 1H), 5.62 (dd, *J* = 6.4, 15.6 Hz, 1H), 5.80 (d, *J* = 15.6 Hz, 1H), 6.48 (d, *J* = 6.4 Hz, 1H), 7.11–7.34 (m, 5H), 7.40–7.44 (m, 4H), 7.75–7.78 (m, 1H), 7.85–7.88 (m, 1H); ^{13}C NMR (100 MHz, $CDCl_3$, Me_4Si) δ 29.46, 33.01, 60.73, 106.32, 117.36, 119.91, 123.41, 124.62, 125.56, 125.87, 126.48, 126.94, 127.14, 128.64, 128.68, 134.19, 142.20, 142.65, 143.99; HRMS–EI: calcd for $C_{23}H_{25}N$: 315.1987, found: 315.1988.

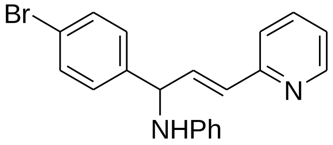

5k

***(E)-N*-(1-(4-Bromophenyl)-3-(pyridin-2-yl)allyl)benzenamine (5k).** Purification of the crude product by flash chromatography on silica gel (eluent: petroleum ether/ethyl acetate = 10/1) afforded the title compound as a red oil in 81% yield. ^{1}H NMR (400 MHz, $CDCl_3$, Me_4Si) δ 4.23 (bs, 1H), 5.09 (d, *J* = 6.0 Hz, 1H), 6.56–6.59 (m, 2H), 6.62 (dd, *J* = 15.6, 1.2 Hz, 1H), 6.68–6.72 (m, 1H), 6.89 (dd, *J* = 6.0, 15.6 Hz, 1H), 7.08–7.21 (m, 4H), 7.28–7.31 (m, 2H), 7.43–7.47 (m, 2H), 7.55–7.60 (m, 1H), 8.51–8.53 (m, 1H); ^{13}C NMR (100 MHz, $CDCl_3$, Me_4Si) δ 59.61, 113.52, 117.91, 121.30, 122.01, 122.37, 128.87, 129.12, 131.01, 131.85, 134.43, 136.49, 140.58, 146.69, 149.48, 154.64; HRMS–ESI: calcd for $C_{20}H_{18}BrN_2$ $[M + H]^+$: 365.0653, found: 365.0638.

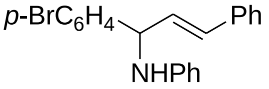

5l

***(E)-N*-(1-(4-Bromophenyl)-3-phenylallyl)benzenamine (5l).** The reaction was carried out on a 2 mmol scale. Purification of the crude product by recycling preparative HPLC afforded the title compound as a colorless oil in 81% yield. ^{1}HNMR (400 MHz, $CDCl_3$, Me_4Si) δ 4.06 (bs, 1H), 5.01 (d, *J* = 6.4 Hz, 1H), 6.31 (dd, *J* = 6.4, 16.0 Hz, 1H), 6.53–6.58 (m, 3H), 6.70 (t, *J* = 7.2 Hz, 1H), 7.10–7.14 (m, 2H), 7.17–7.34 (m, 7H), 7.44–7.46 (m, 2H); ^{13}C NMR (100 MHz, $CDCl_3$, Me_4Si) δ 60.03, 113.59, 117.94, 121.21, 126.49, 127.85, 128.56, 128.84, 129.15, 130.02, 131.62, 131.84, 136.28, 141.04, 146.81; HRMS–EI: calcd for $C_{21}H_{18}NBr$: 363.0623, found: 363.0621.

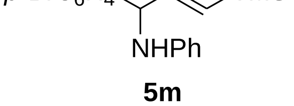

5m

***(E)-N*-(1-(4-Bromophenyl)-3-(trimethylsilyl)allyl)benzenamine (5m).** Purification of the crude product by preparative TLC on silica gel (eluent: petroleum ether/ethyl acetate = 50/1) afforded the title compound as a white solid in 84% yield. ^{1}H NMR (300 MHz, $CDCl_3$, Me_4Si) δ 0.20 (s, 9H), 4.19 (bs, 1H), 5.01 (d, *J* = 5.1 Hz, 1H), 6.02 (d, *J* = 18.6 Hz,

1H), 6.27 (dd, J = 5.4, 18.6 Hz, 1H), 6.68 (d, J = 8.4 Hz, 2H), 6.83 (t, J = 7.2 Hz, 1H), 7.25 (dd, J = 7.8, 7.2 Hz, 2H), 7.36 (d, J = 8.1 Hz, 2H), 7.59 (d, J = 8.1 Hz, 2H); ^{13}C NMR (75.5 MHz, $CDCl_3$, Me_4Si) δ −1.37, 62.12, 113.51, 117.73, 121.01, 128.85, 129.09, 131.67, 131.76, 141.01, 145.42, 146.87. HRMS–EI: calcd for $C_{18}H_{22}NBrSi$: 359.0705, found: 359.0702.

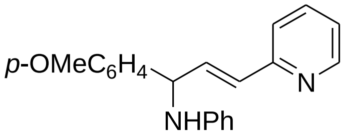

5n

***(E)*-*N*-(1-(4-Methoxyphenyl)-3-(pyridin-2-yl)allyl)benzenamine (5n).** Purification of the crude product by flash chromatography on silica gel (eluent: petroleum ether/ethyl acetate = 5/1) afforded the title compound as a colorless oil in 75% yield. ^{1}H NMR (400 MHz, $CDCl_3$, Me_4Si) δ 3.78 (s, 3H), 4.15 (bs, 1H), 5.08 (d, J = 6.0 Hz, 1H), 6.61–6.71 (m, 4H), 6.87 (d, J = 8.4 Hz, 2H), 6.90–6.94 (m, 1H), 7.08–7.15 (m, 3H), 7.21–7.24 (m, 1H), 7.40 (d, J = 8.4 Hz, 2H), 7.57 (dt, J = 2.0, 7.2 Hz, 1H), 8.51-8.53 (m, 1H); ^{13}C NMR (100 MHz, $CDCl_3$, Me_4Si) δ 55.24, 59.61, 113.50, 114.17, 117.60, 121.87, 122.14, 128.40, 129.09, 130.31, 133.61, 135.35, 136.43, 147.15, 149.47, 155.12, 159.00; HRMS–EI: calcd for $C_{21}H_{20}N_2O$: 316.1576, found: 316.1572.

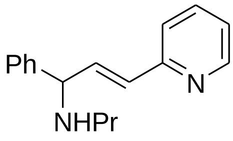

5o

***(E)*-1-Phenyl-*N*-propyl-3-(pyridin-2-yl)prop-2-en-1-amine (5o).** Purification of the crude product by flash chromatography on silica gel (eluent: petroleum ether/ethyl acetate = 5/1) afforded the title compound as a colorless oil in 60% yield. ^{1}H NMR (400 MHz, $CDCl_3$, Me_4Si) δ 0.91 (t, J = 7.2 Hz, 3H), 1.49–1.58 (m, 2H), 1.75 (bs, 1H) 2.49–2.66 (m, 2H), 4.41 (d, J = 7.2 Hz, 1H), 6.67 (d, J = 16.0 Hz, 1H), 6.77 (dd, J = 7.2, 16.0 Hz, 1H), 7.06–7.10 (m, 1H), 7.24–7.35 (m, 4H), 7.39–7.42 (m, 2H), 7.56–7.60 (m, 1H), 8.50–8.52 (m, 1H); ^{13}C NMR (100 MHz, $CDCl_3$, Me_4Si) δ 11.76, 23.23, 49.62, 65.23, 121.27, 121.93, 127.24, 127.32, 128.56, 129.91, 136.34, 137.25, 142.59, 149.38, 155.49; HRMS–EI: calcd for $C_{17}H_{20}N_2$: 252.1626, found: 252.1623.

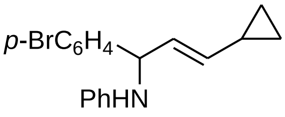

5q

(*E*)-*N*-(1-(4-Bromophenyl)-3-cyclopropylallyl)benzenamine (5q). After the reaction was finished, the mixture was quenched by saturated aqueous $NaHCO_3$ solution and stirred for ca. 1–2 h at room temperature. The resulting mixture was extracted with diethyl ether three times. The combined extract was washed separately with brine, and dried over anhydrous Na_2SO_4. The solvent was evaporated in vacuo and the residue was detected by NMR. NMR yield: 95%. ^{1}H NMR (400 MHz, $CDCl_3$, Me_4Si) δ 0.32–0.35 (m, 2H), 0.67–0.71 (m, 2H), 1.32–1.41 (m, 1H), 4.00 (br, 1H), 4.79 (d, *J* = 6.8 Hz, 1H), 5.10 (dd, *J* = 8.8, 15.2 Hz, 1H), 5.66 (dd, *J* = 6.8, 15.2 Hz, 1H), 6.49 (d, *J* = 8.0 Hz, 2H), 6.67 (t, *J* = 7.2 Hz, 1H), 7.09 (dd, *J* = 7.2, 8.0 Hz, 2H), 7.22 (d, *J* = 8.4 Hz, 2H), 7.42 (d, *J* = 8.4 Hz, 2H); ^{13}C NMR (100 MHz, $CDCl_3$, Me_4Si) δ 6.79, 6.80, 13.53, 59.75, 113.45, 117.61, 120.75, 128.35, 128.62, 129.04, 131.63, 137.08, 141.69, 146.88; HRMS–EI: calcd for $C_{18}H_{18}BrN$: 327.0623, found: 327.0620.

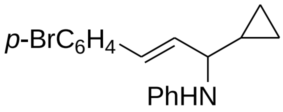

6q

(*E*)-*N*-(3-(4-Bromophenyl)-1-cyclopropylallyl)benzenamine (6q). Purification of the crude product by preparative TLC on silica gel (eluent: petroleum ether/ethyl acetate = 50/1) afforded the title compound as a colorless oil in 74% yield. ^{1}HNMR (300 MHz, $CDCl_3$, Me_4Si) δ 0.37–0.42 (m, 2H), 0.56–0.63 (m, 2H), 1.03–1.10 (m, 1H), 3.28–3.33 (m, 1H), 4.03 (bs, 1H), 6.22 (dd, *J* = 5.7, 15.6 Hz, 1H), 6.53 (d, *J* = 15.9 Hz, 1H), 6.61 (d, *J* = 7.8 Hz, 2H), 6.68 (t, *J* = 7.2 Hz, 1H), 7.14 (dd, *J* = 7.5, 8.4 Hz, 2H), 7.21 (d, *J* = 8.4 Hz, 2H), 7.40 (d, *J* = 8.4 Hz, 2H); ^{13}C NMR (75.5 MHz, $CDCl_3$, Me_4Si) δ 2.94, 3.55, 17.06, 60.07, 113.30, 117.35, 120.95, 127.89, 128.78, 129.10, 131.50, 131.59, 135.92, 147.63; HRMS–EI: calcd for $C_{18}H_{18}NBr$: 327.0623, found: 327.0618.

Acylation of compound 5l

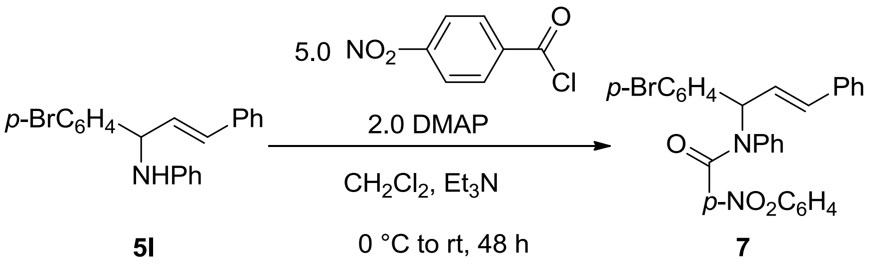

(*E*)-*N*-(1-(4-Bromophenyl)-3-phenylallyl)-4-nitro-*N*-phenylbenzamide (7). To a stirred solution of allylic amine **5l** (182 mg, 0.5 mmol) in CH_2Cl_2 (11 mL) was added Et_3N (3 mL) at 0 °C. Then 4-nitrobenzoyl chloride (2.5 mmol, 464 mg) was added at 0 °C, after the acyl chloride was dissolved, 4-dimethylaminopyridine (120 mg) was added. The mixture was warmed up to room temperature and stirred for 48 h. The resulting mixture was quenched by saturated NH_4Cl (5 mL), and extracted with dichloromethane three times. The combined extract was washed separately with water and brine, and dried over anhydrous Na_2SO_4. The solvent was evaporated in vacuo and the residue was purified by column chromatography on silica gel (eluent: petroleum ether/ethyl acetate = 20/1 to 10/1) to afford the desired compound as a red solid (210 mg, 82% isolated yield). ^{1}H NMR (400 MHz, $CDCl_3$, Me_4Si) δ 6.43 (dd, *J* = 8.8, 16.0 Hz, 1H), 6.65 (d, *J* = 8.8 Hz, 1H), 6.81 (d, *J* = 16.0 Hz, 1H), 6.79–6.84 (m, 2H), 7.13–7.16 (m, 3H), 7.24–7.38 (m, 7H), 7.42–7.48 (m, 4H), 7.97 (d, *J* = 8.8 Hz, 2H); ^{13}C NMR (100 MHz, $CDCl_3$, Me_4Si) δ 62.04, 121.76, 122.89, 124.65, 126.60, 128.16, 128.20, 128.60, 129.09, 129.15, 129.68, 129.96, 131.55, 135.09, 136.05, 138.48, 139.50, 142.26, 147.67, 168.23; HRMS–EI: calcd for $C_{28}H_{21}BrN_2O_3$: 512.0736, found: 512.0731.

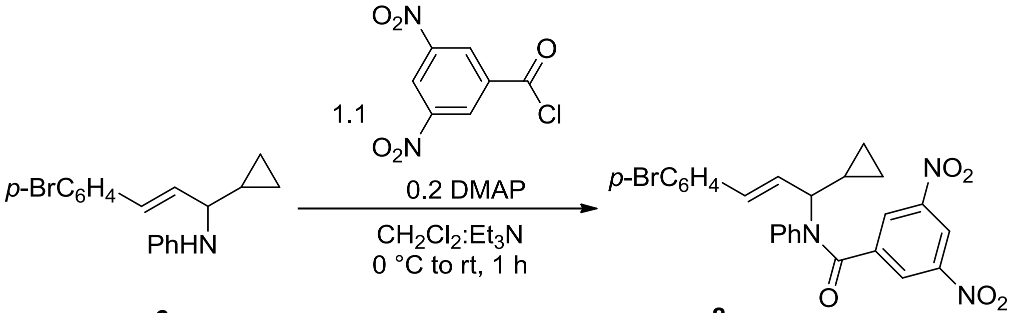

6q **8**

***(E)*-*N*-(3-(4-Bromophenyl)-1-cyclopropylallyl)-3,5-dinitro-*N*-phenylbenzamide (8).** To a stirred solution of allylic amine **6q** (351 mg, 1.07 mmol) in CH_2Cl_2 (3.0 mL) was added dropwise Et_3N (0.32 mL, 2.3 mmol) at 0 °C. And then 3,5-dinitrobenzoyl chloride (272 mg, 1.18 mmol) in 2 mL CH_2Cl_2 was added at this temperature. 4-Dimethylaminopyridine (24 mg, 0.2 mmol) was added and then the mixture was warmed up to room temperature. After stirring for 1 h, the resulting mixture was quenched by saturated NH_4Cl (5 mL), and extracted with dichloromethane three times. The combined extract was washed separately with water and brine, and dried over anhydrous Na_2SO_4. The solvent was evaporated in vacuo and the residue was purified twice by column chromatography on silica gel to afford the desired compound as a white solid (334 mg, 64% isolated yield). ^{1}H NMR (400 MHz, $CDCl_3$, Me_4Si) δ 0.47–0.53 (m, 1H), 0.63–0.69 (m, 2H), 0.73–0.80 (m, 1H), 0.95–1.04 (m, 1H), 4.74–4.78 (m, 1H), 6.37 (dd, *J* = 7.2, 16.0 Hz, 1H), 6.68 (d, *J* = 16.0 Hz, 1H), 7.20–7.32 (m, 7H), 7.44–7.47 (m, 2H), 8.44 (d, *J* = 2.0 Hz, 2H), 8.83 (t, *J* = 2.4 Hz, 1H); ^{13}C NMR (100 MHz, $CDCl_3$, Me_4Si) δ 4.33, 5.84, 13.48, 64.23, 119.10, 121.77, 127.72, 128.06, 128.52, 128.82, 129.57, 130.10, 131.71, 131.81, 135.36, 138.93, 139.88, 147.66, 165.36; HRMS–EI: calcd for $C_{25}H_{20}BrN_3O_5$: 521.0586, found: 521.0585.

Procedure for the iodoallylic amine derivative 9

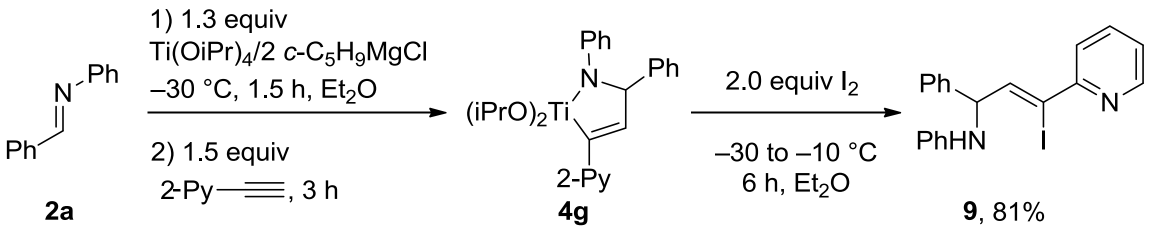

2a **4g** **9**, 81%

***(Z)*-*N*-(3-Iodo-1-phenyl-3-(pyridin-2-yl)allyl)benzenamine (9).** To a stirred solution of imine **2a** (91 mg, 0.5 mmol) and $Ti(OiPr)_4$ (0.19 mL, 0.65 mmol) in Et_2O (5 mL) was added dropwise *c*-C_5H_9MgCl (0.65 mL, 2.0 M solution in diethyl ether, 1.3 mmol) at –30 °C. The solution was stirred at this temperature for 1.5 h, and then 2-ethynylpyridine (76 µL, 77 mg, 0.75 mmol) was added into the reaction mixture at –30 °C. After stirring for 3 h at –30 °C, iodine (254 mg, 1 mmol) was added and then warmed up to –10 °C. After stirring for 6 h at this temperature, saturated aqueous $Na_2S_2O_3$ (5 mL) solution was added to quench the reaction and was stirred overnight. The resulting mixture was extracted with diethyl ether three times. The combined extract was washed with brine, and dried over anhydrous Na_2SO_4. The solvent was evaporated in vacuo and the residue was purified by column chromatography on silica gel (petroleum ether/ethyl acetate = 30/1 as eluent) to afford the desired allylic amine **9** as a red solid (167 mg, 81% isolated yield). ^1H NMR (400 MHz, $CDCl_3$, Me_4Si) δ 4.30 (bs, 1H), 5.43 (d, *J* = 8.4 Hz, 1H), 6.67 (d, *J* = 7.2 Hz, 2H), 6.73 (t, *J* = 7.2 Hz, 1H), 6.91 (d, *J* = 8.4 Hz, 1H), 7.14–7.19 (m, 3H), 7.31–7.40 (m, 3H), 7.56–7.62 (m, 4H), 8.54–8.56 (m, 1H); ^{13}C NMR (100 MHz, $CDCl_3$, Me_4Si) δ 64.78, 107.23, 113.69, 118.04, 122.74, 123.18, 127.17, 127.91, 128.98, 129.25, 136.76, 140.71, 142.81, 147.02, 149.05, 156.66; HRMS–EI: calcd for $C_{20}H_{17}IN_2$: 412.0437, found: 412.0432.

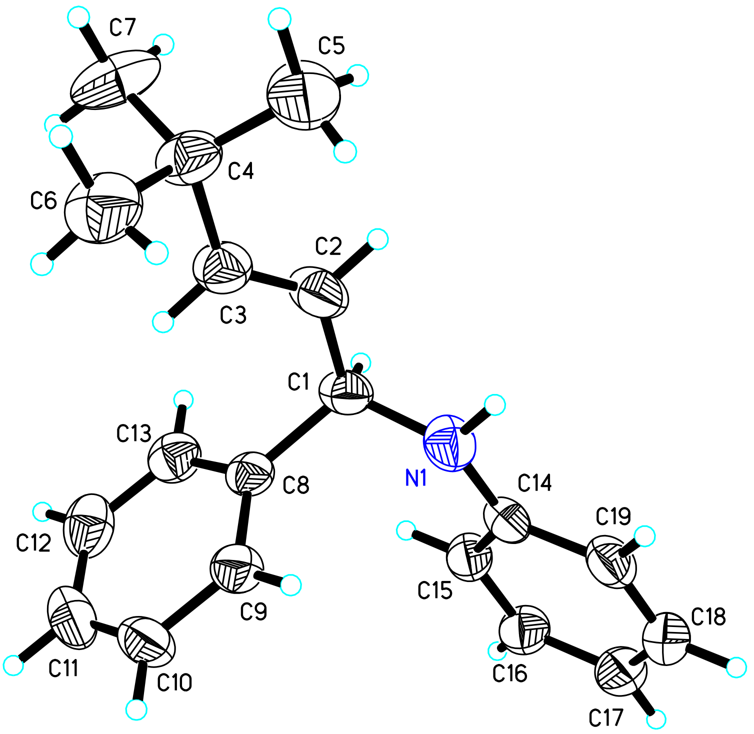

X-ray crystal structure of compound **5c** (methyl groups are disordered)

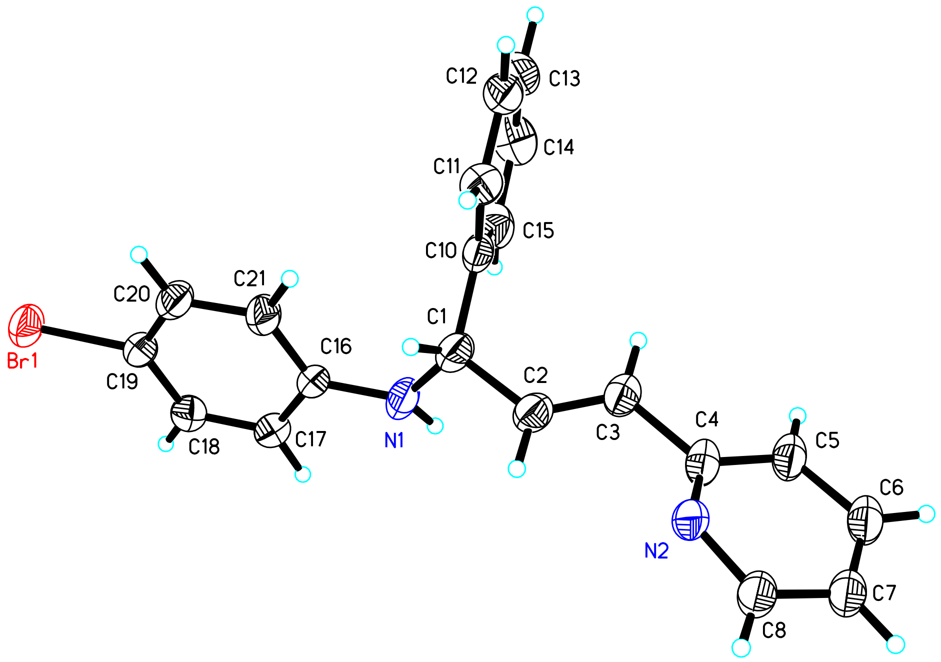

X-ray crystal structure of compound **5h** (several atoms are disordered)

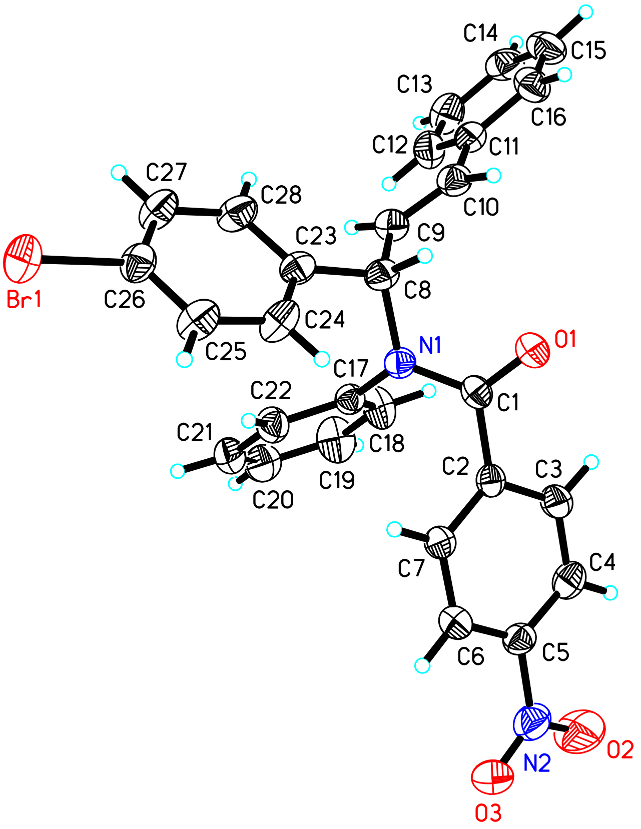

X-ray crystal structure of compound **7**

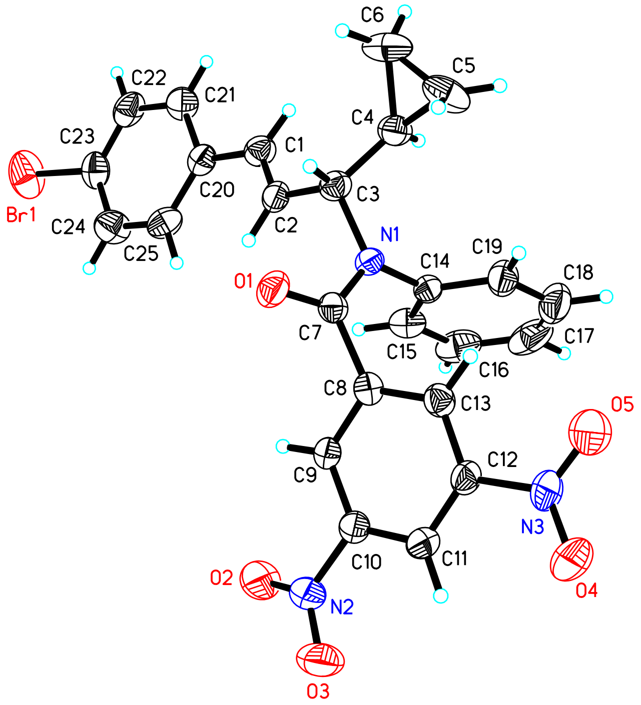

X-ray crystal structure of compound **8**

NMR spectra of 5a

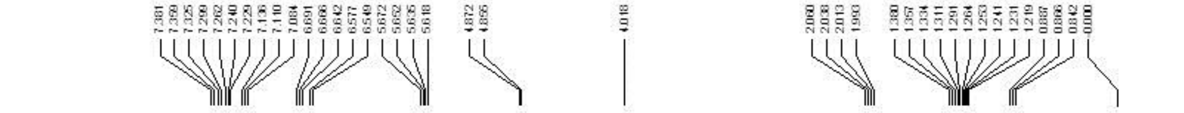

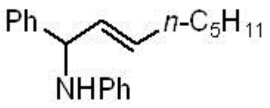

5a

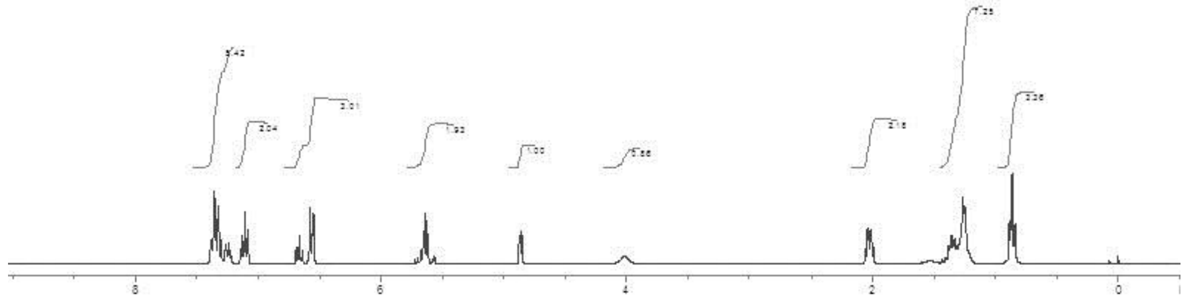

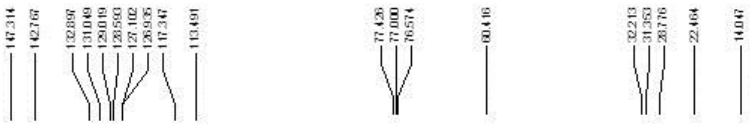

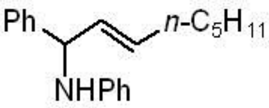

5a

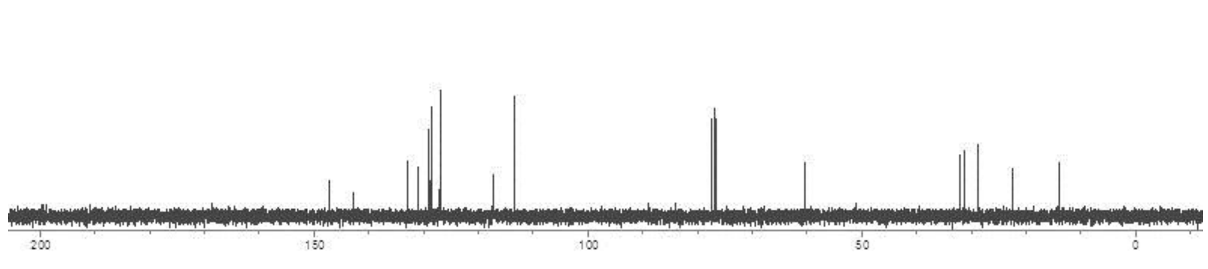

NMR spectra of 5b

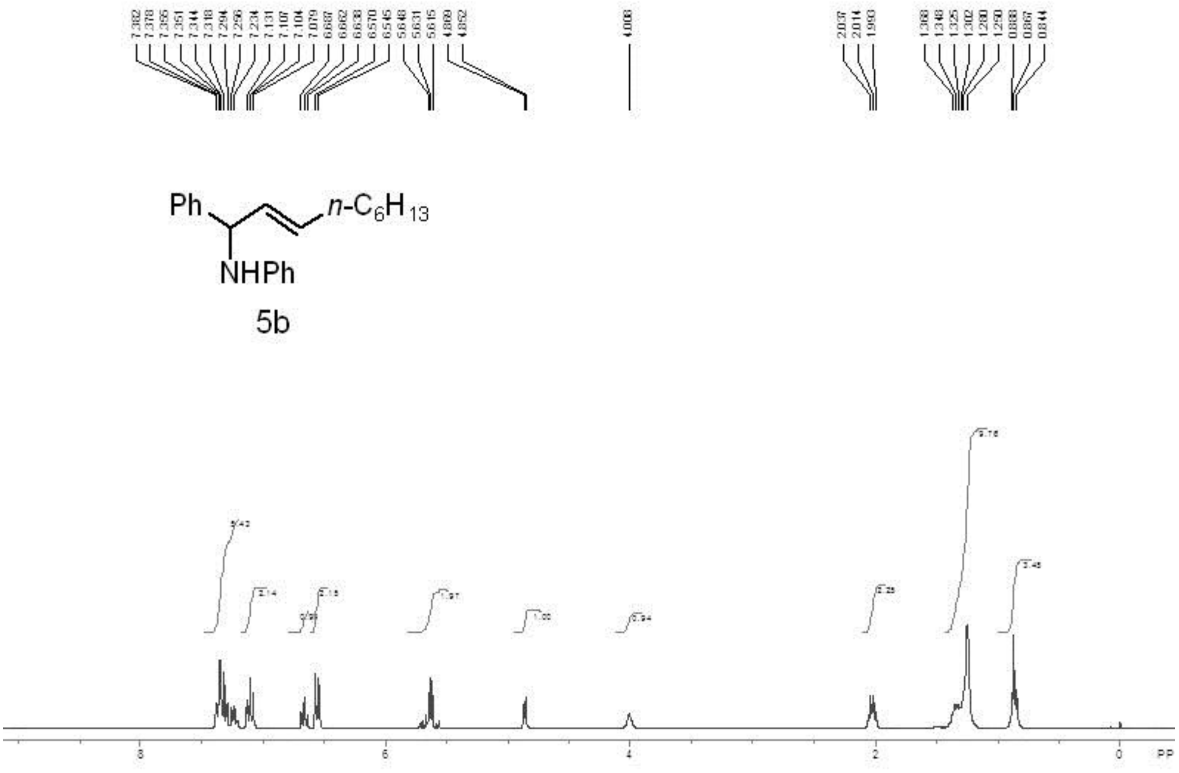

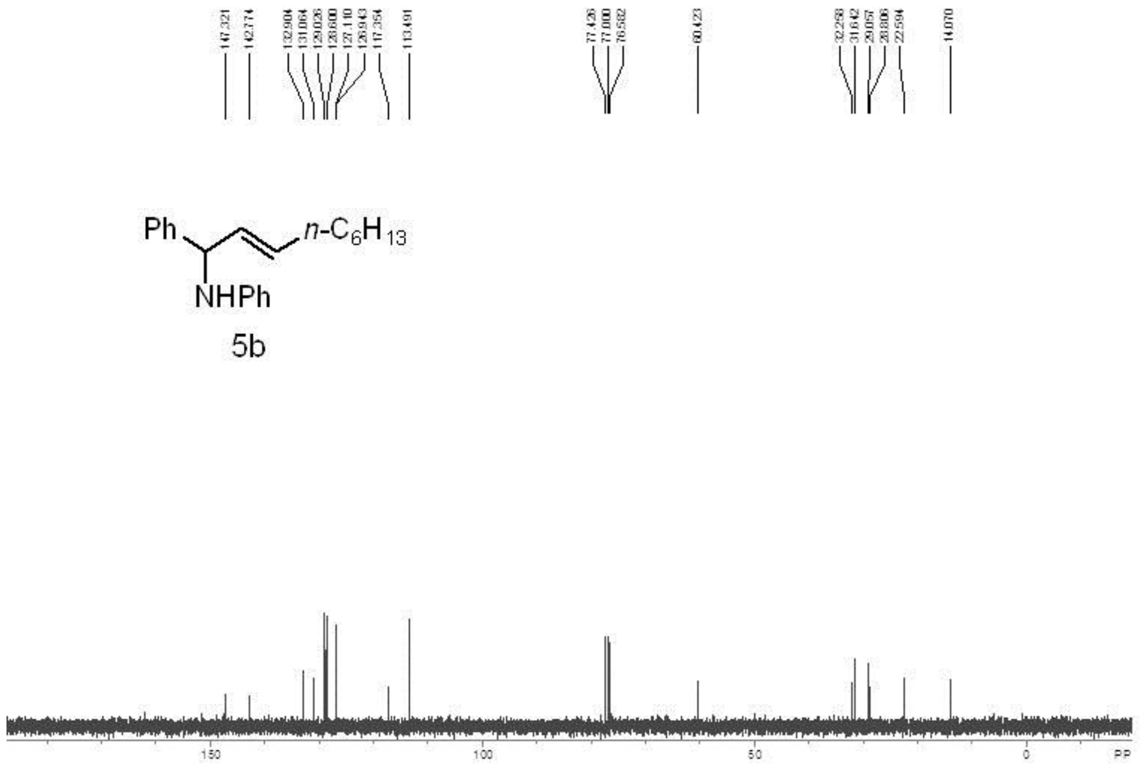

NMR spectra of 5c

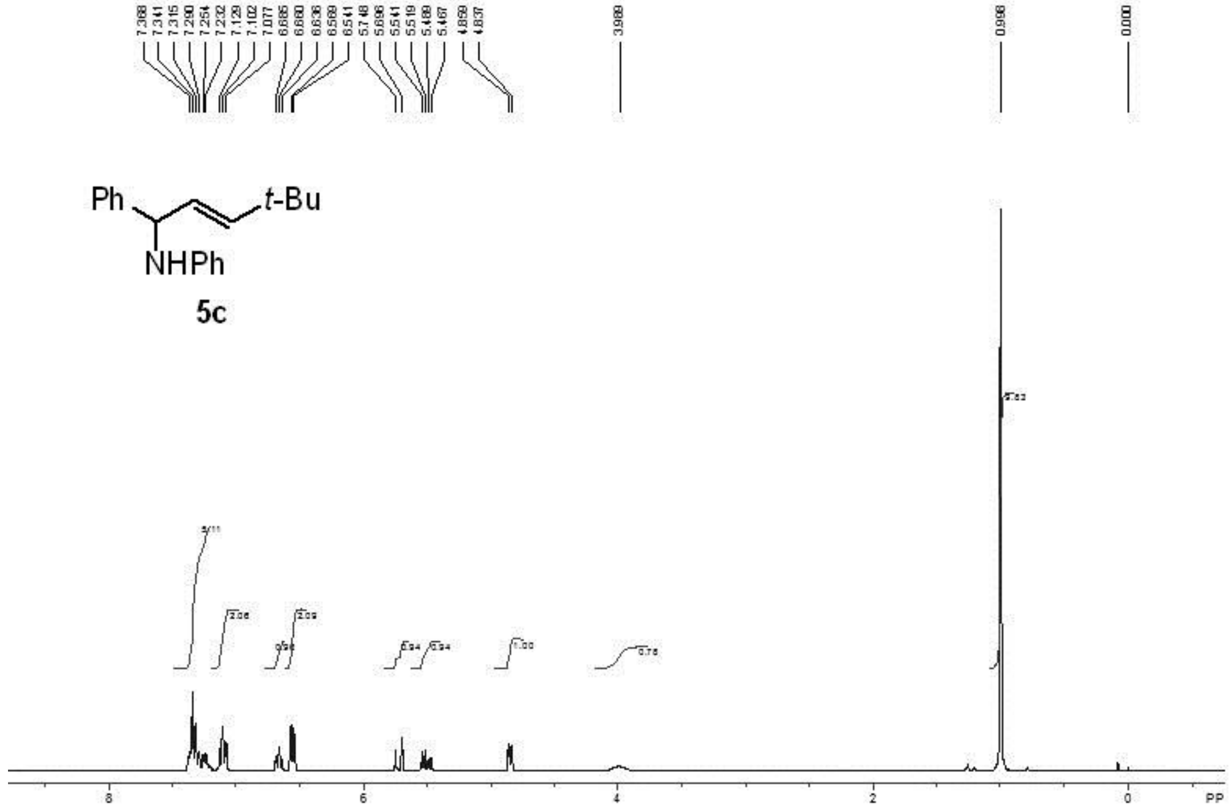

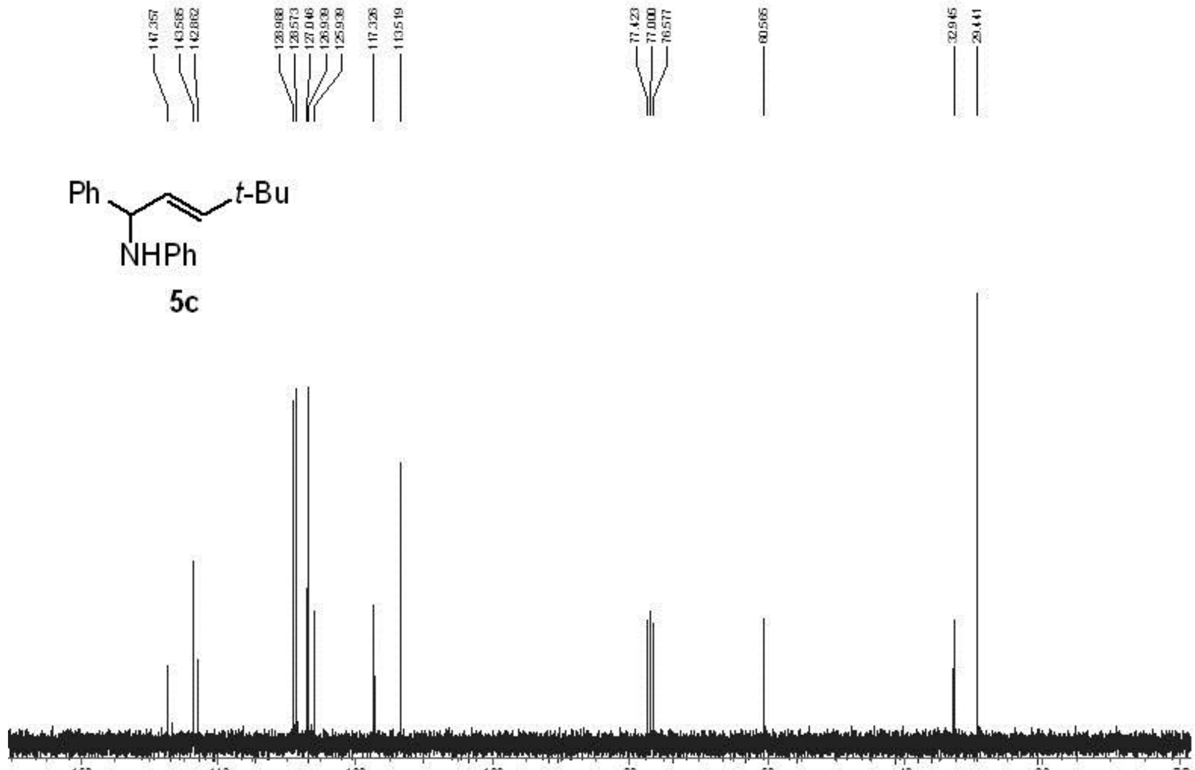

NMR spectra of 5d

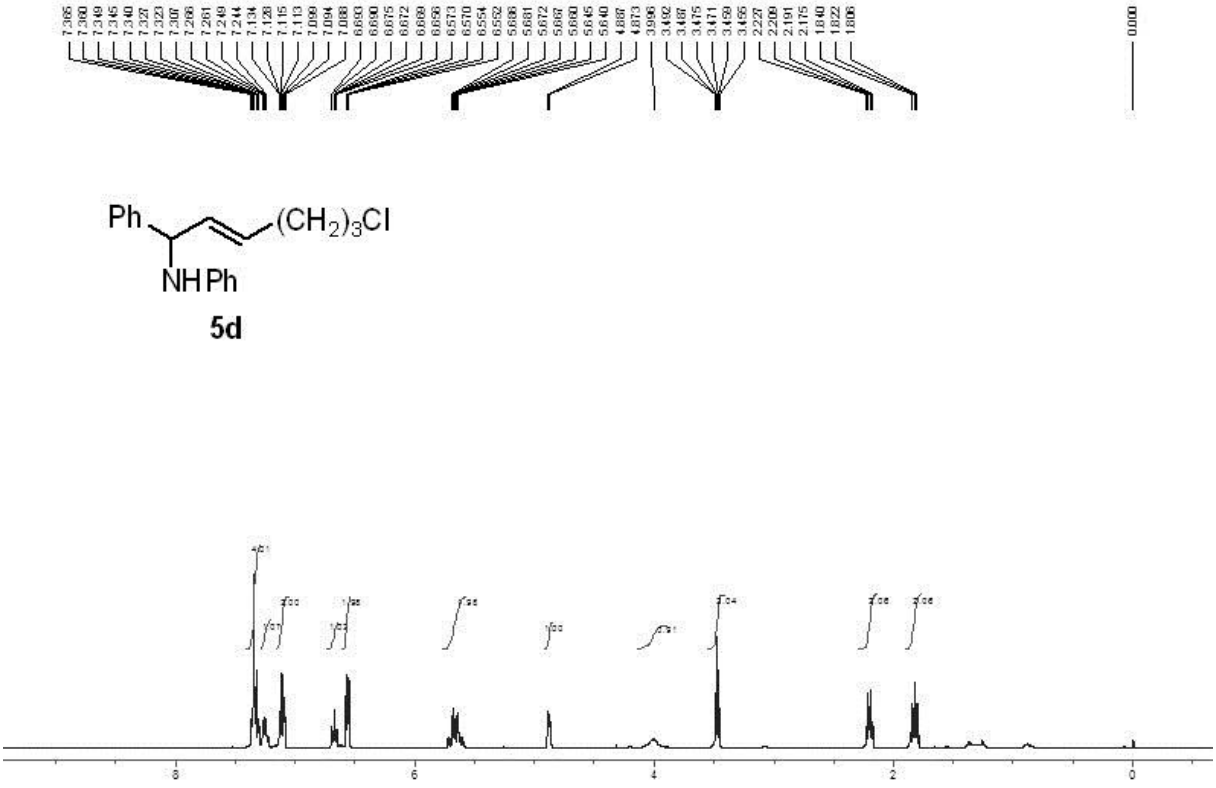

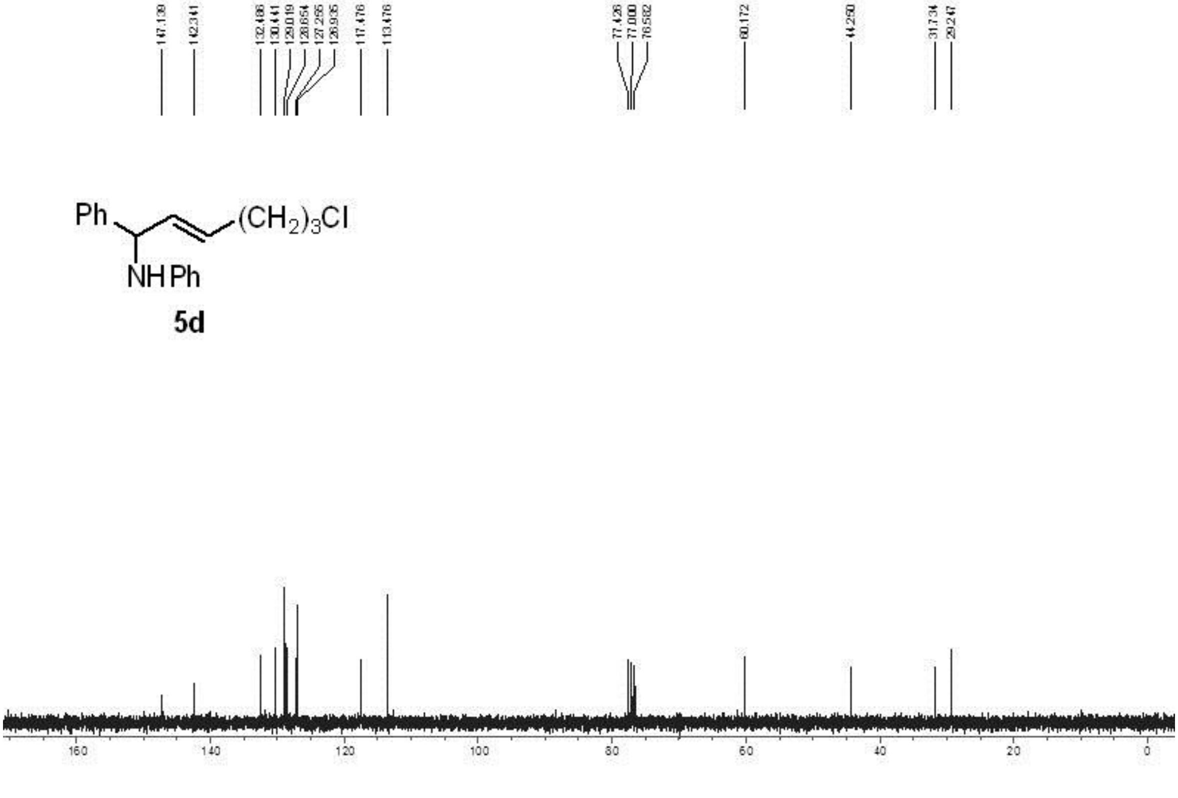

NMR spectra of 5e

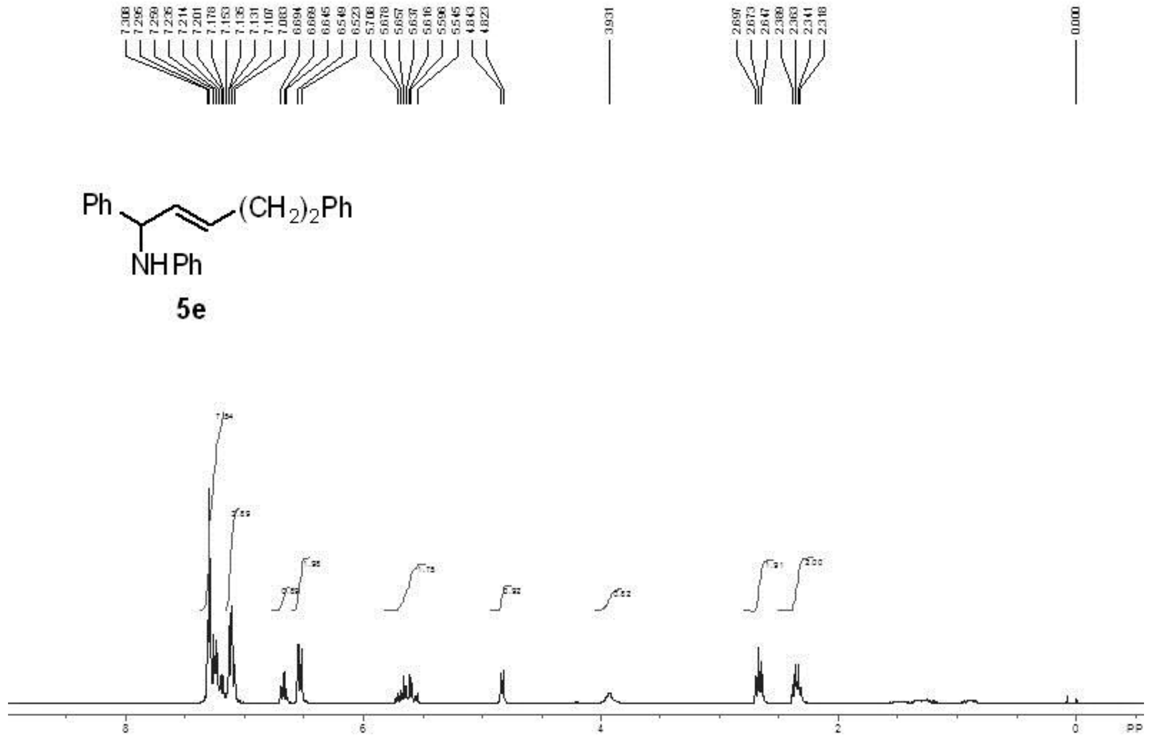

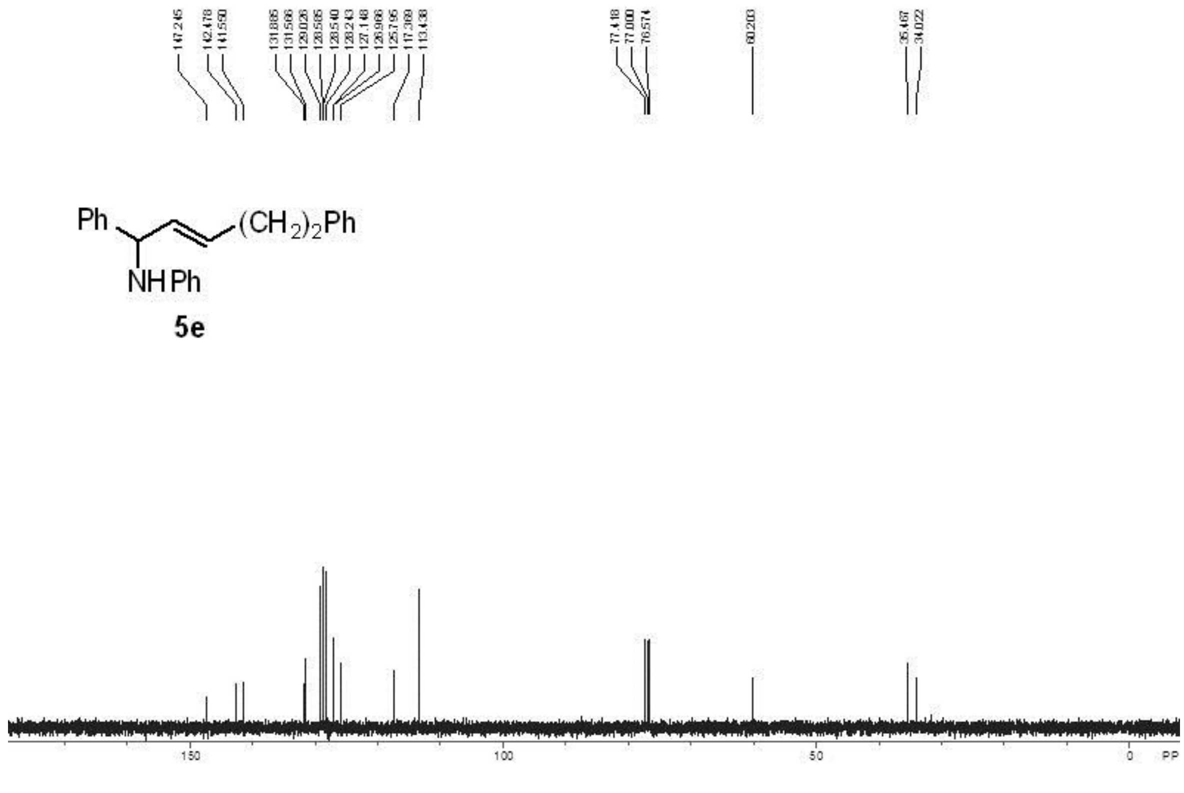

NMR spectra of 5f

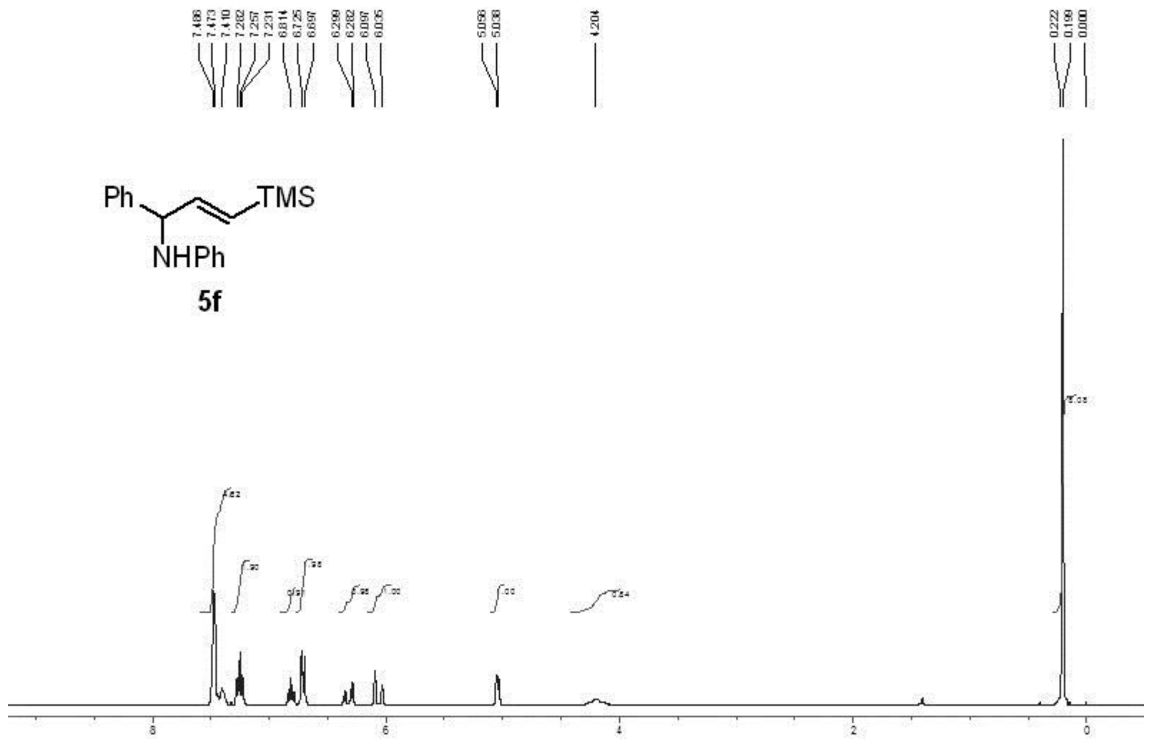

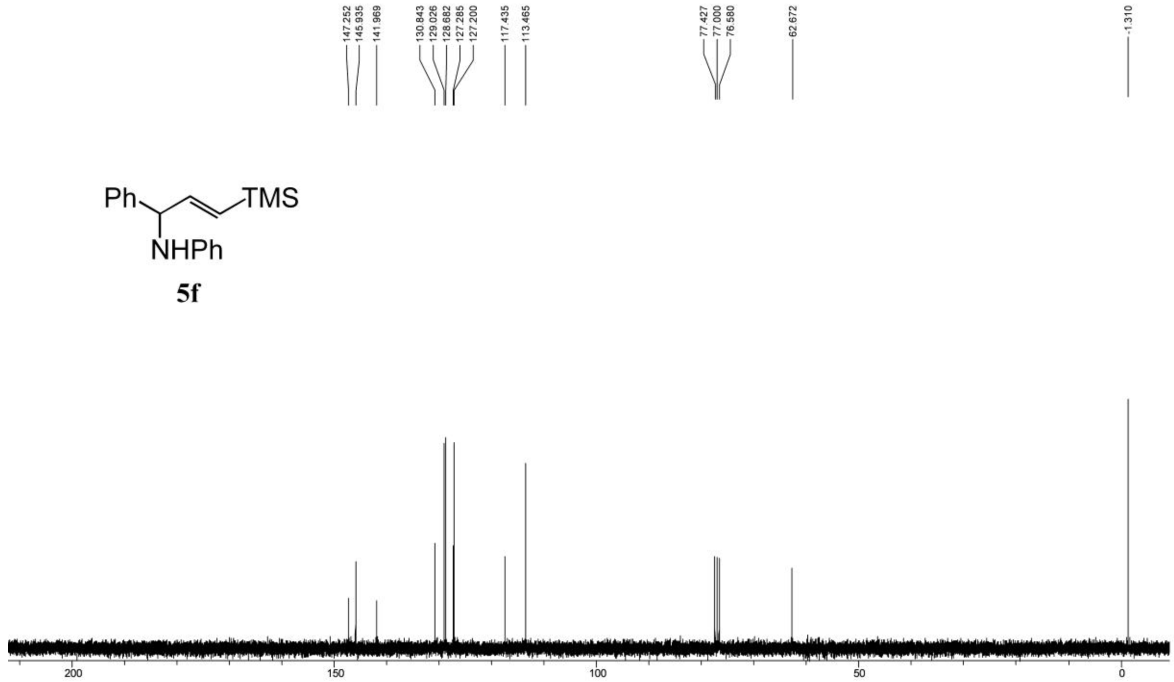

NMR spectra of 5g

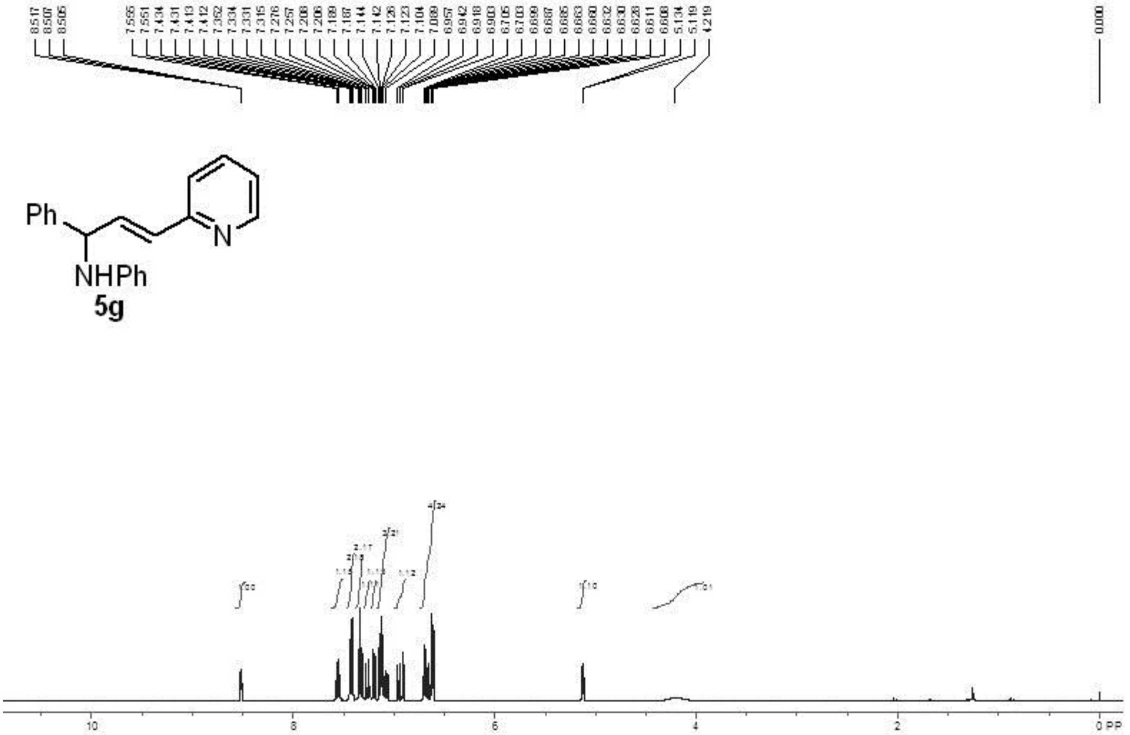

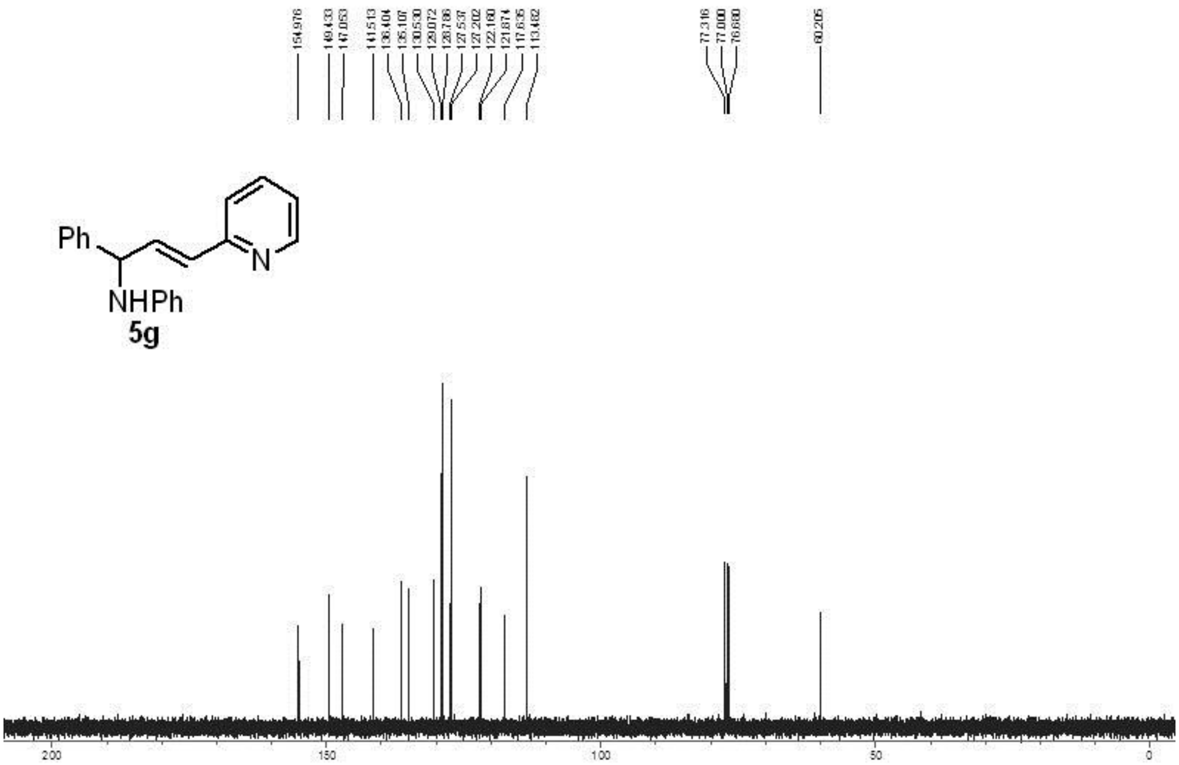

NMR spectra of 5h

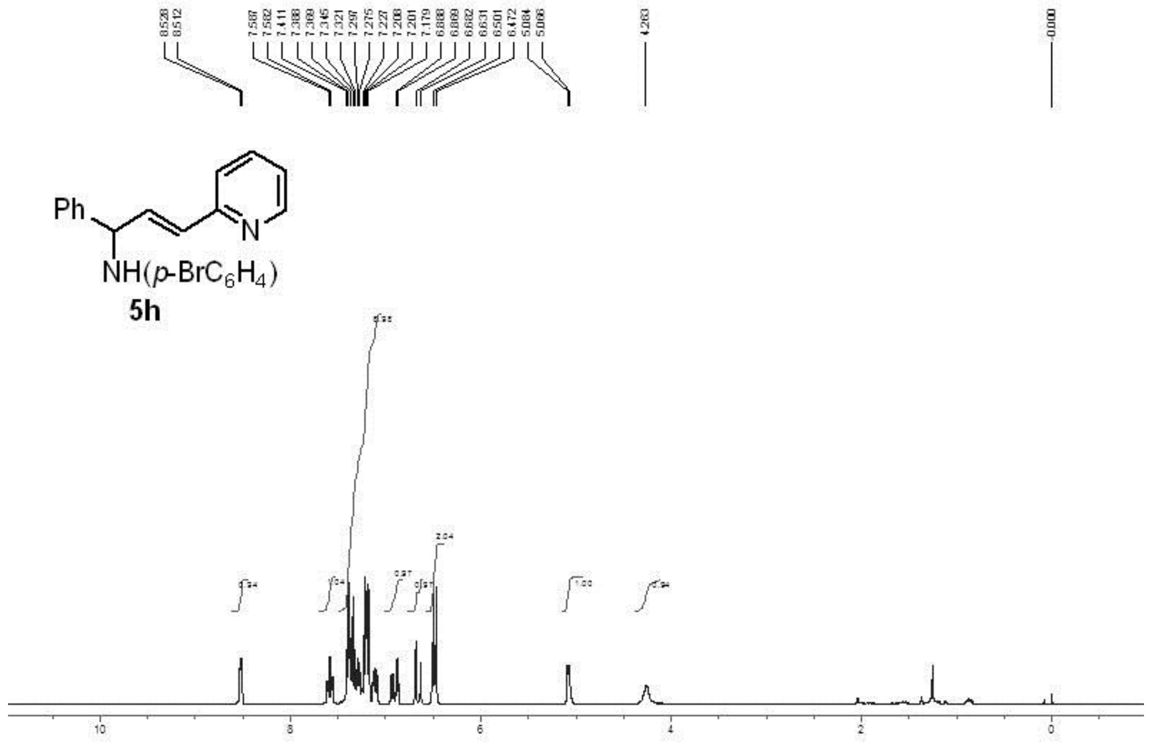

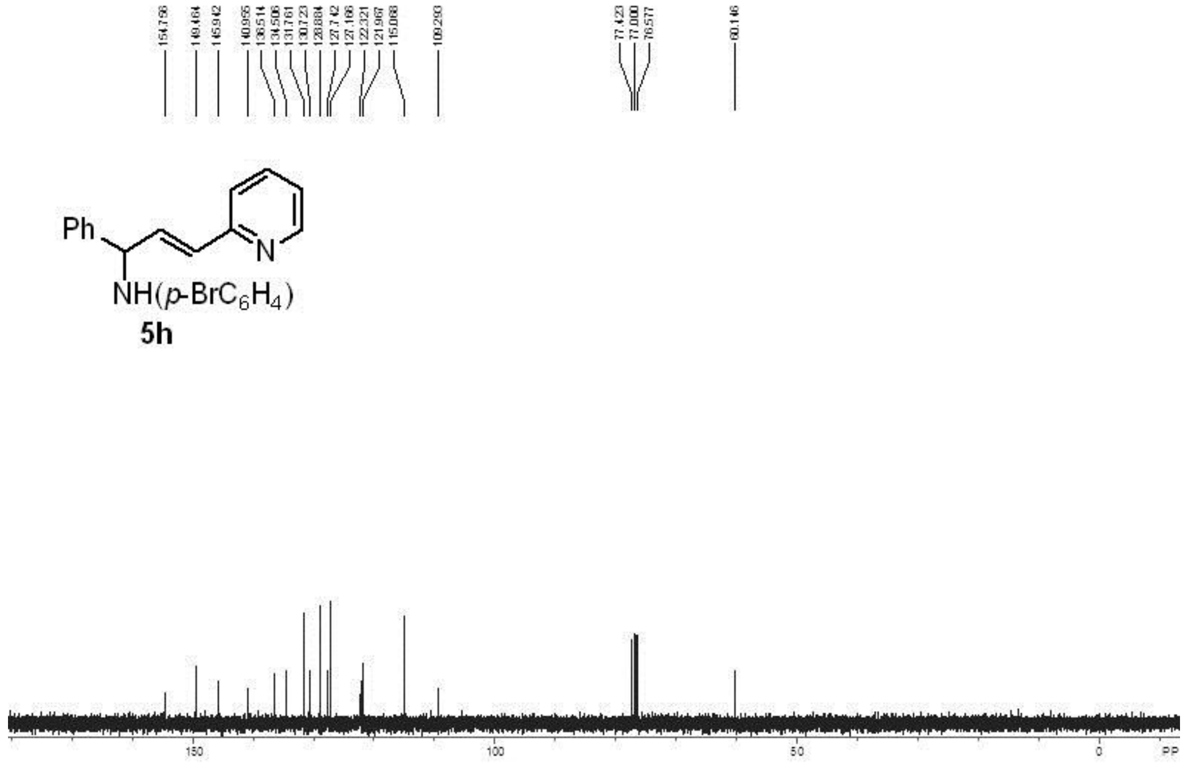

NMR spectra of 5i

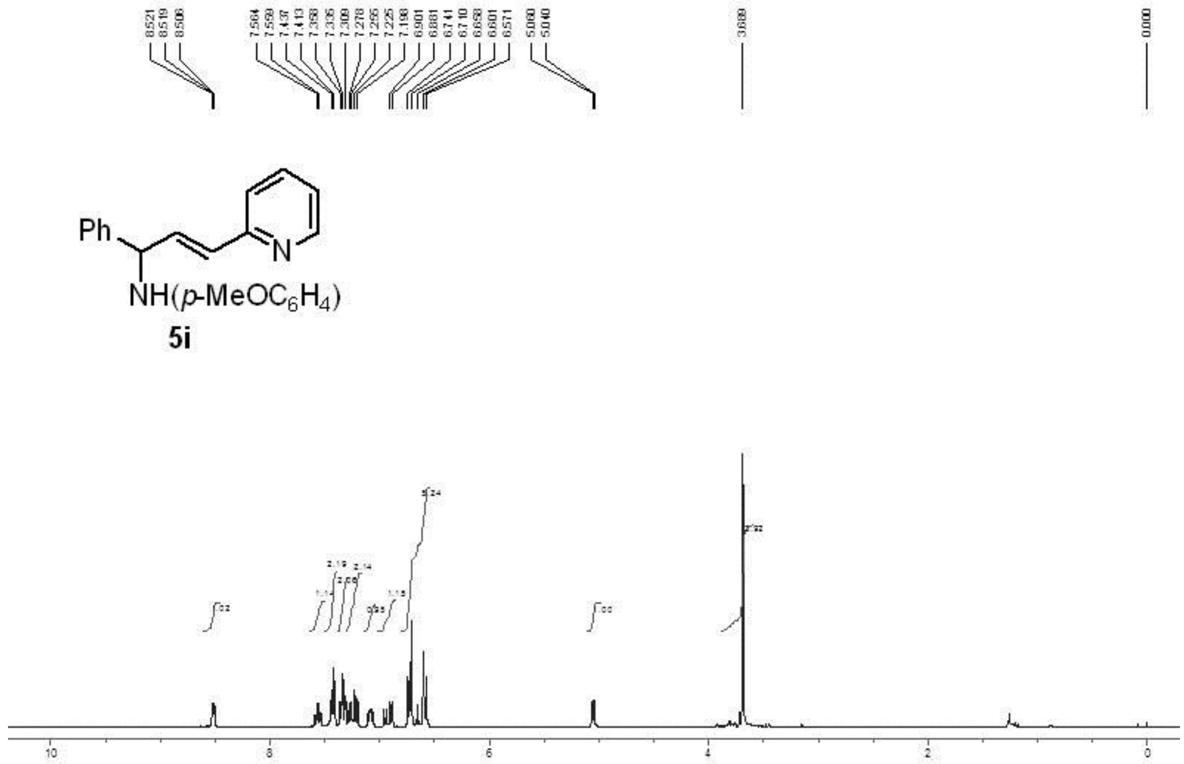

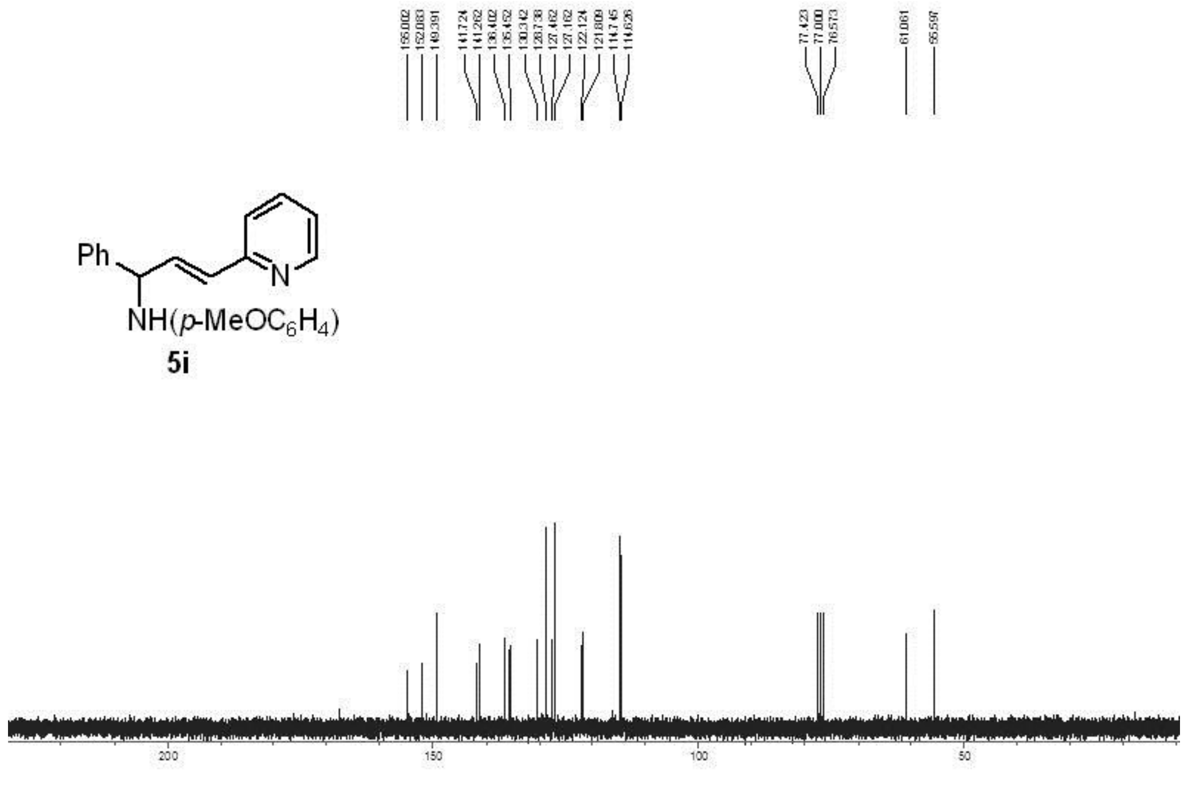

NMR spectra of 5j

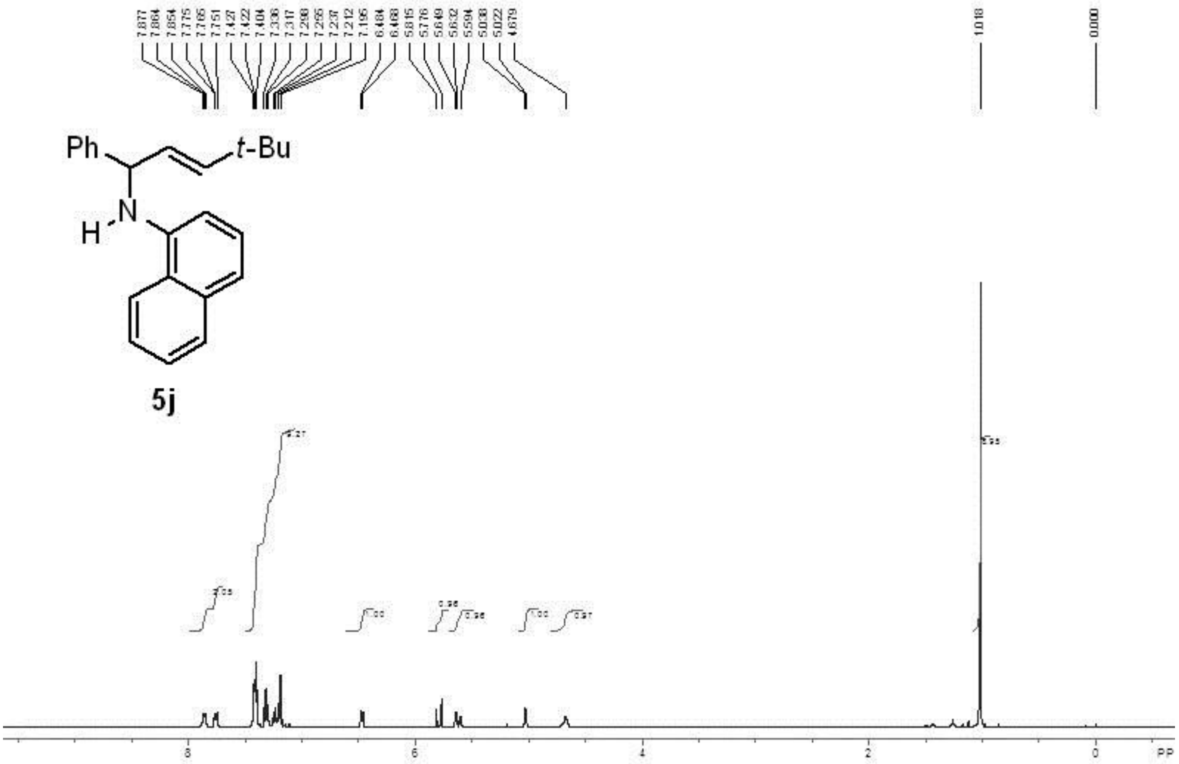

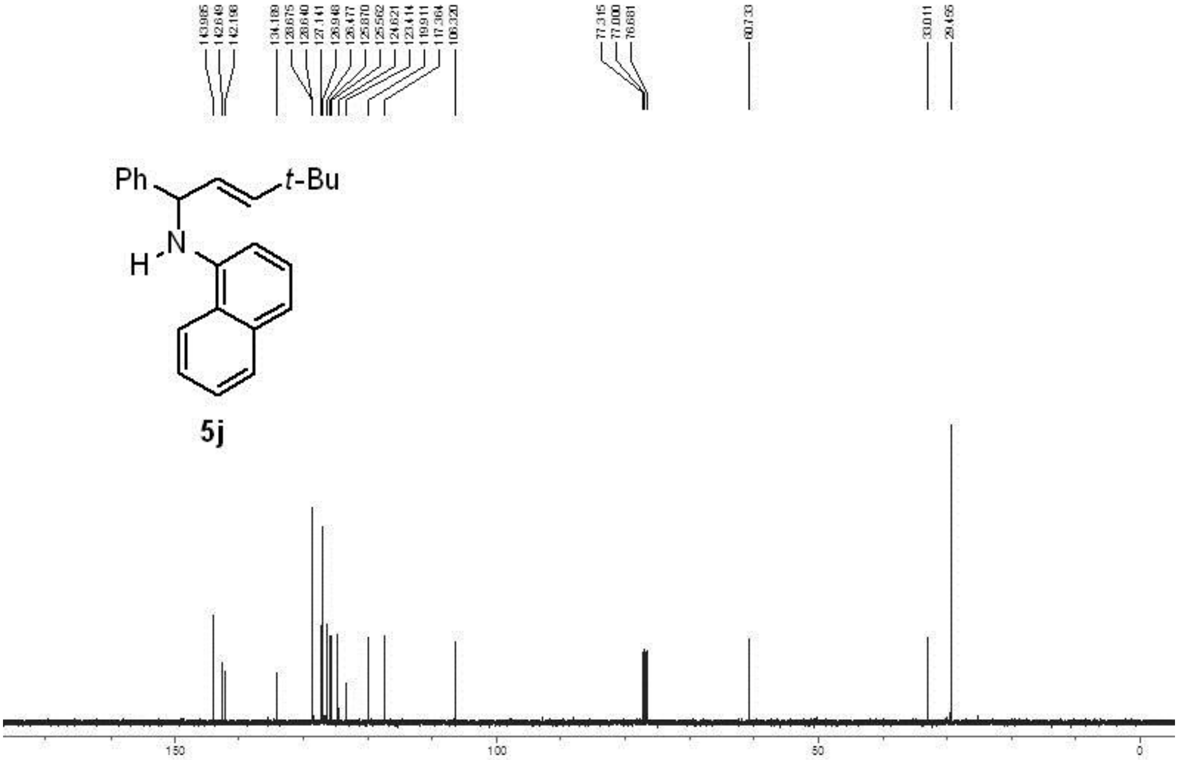

NMR spectra of 5k

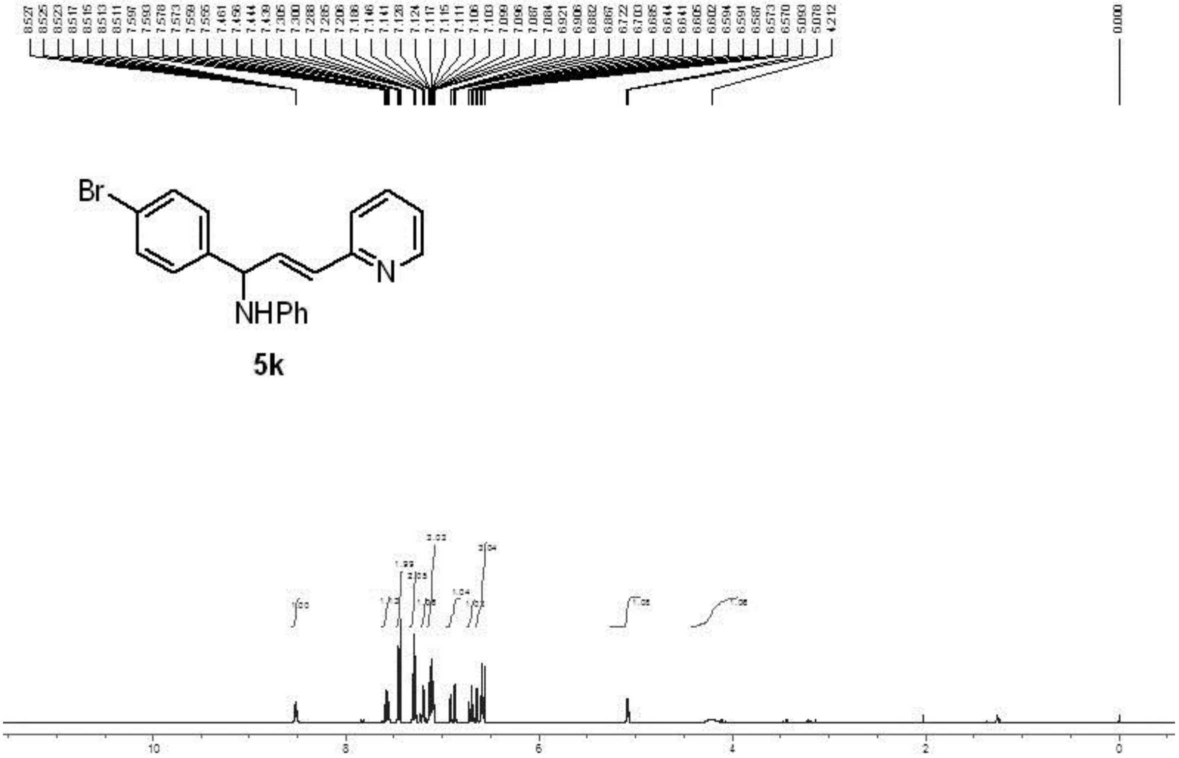

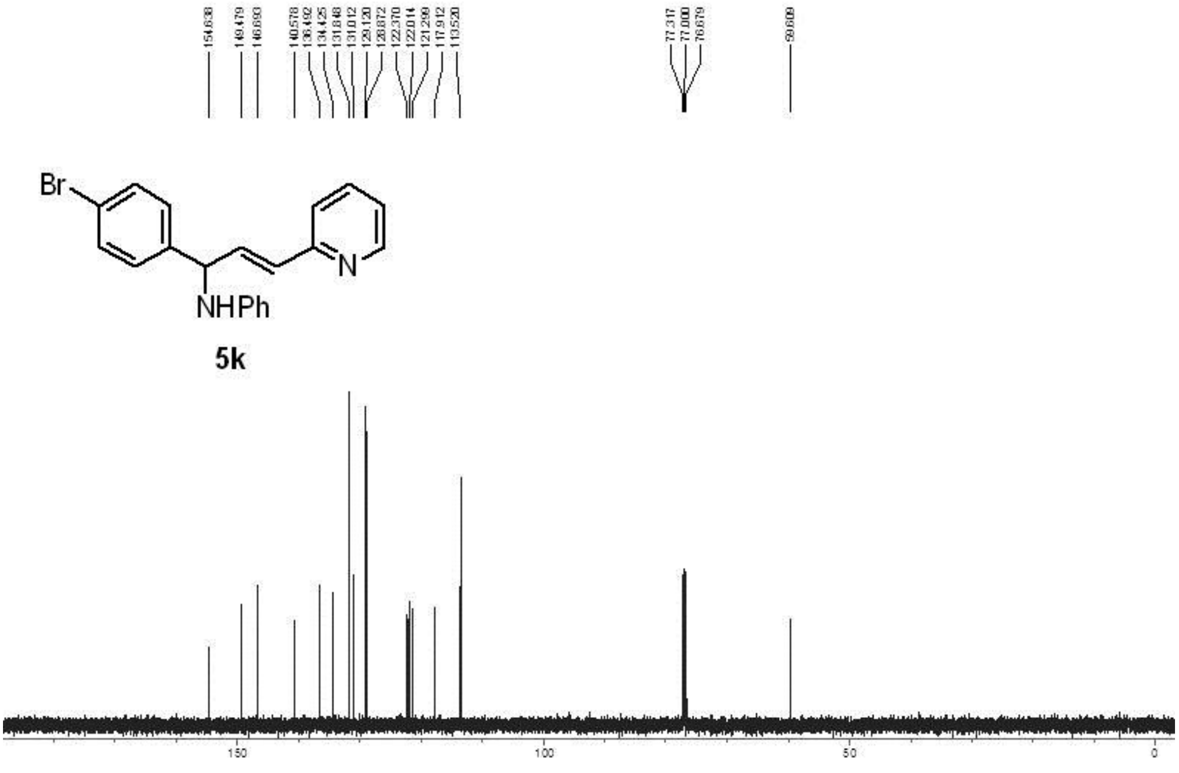

NMR spectra of 5l

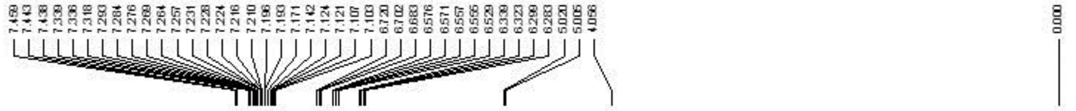

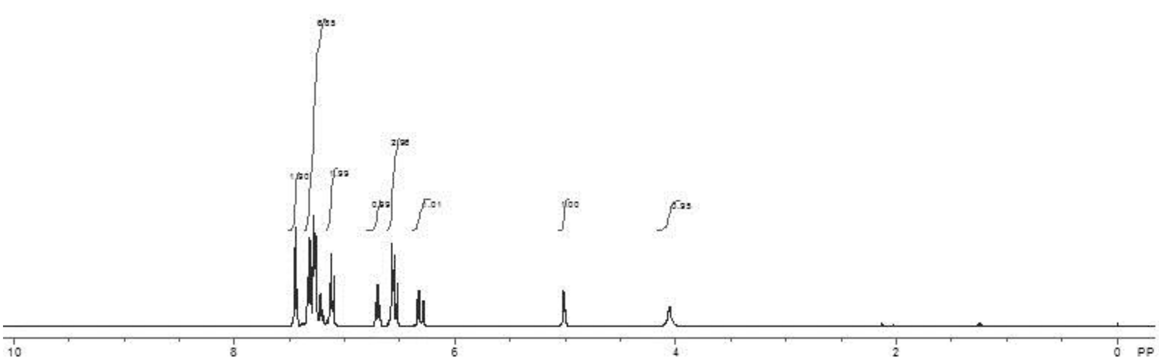

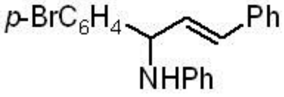

5l

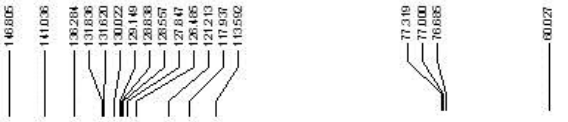

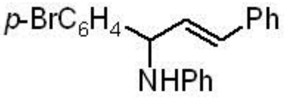

5l

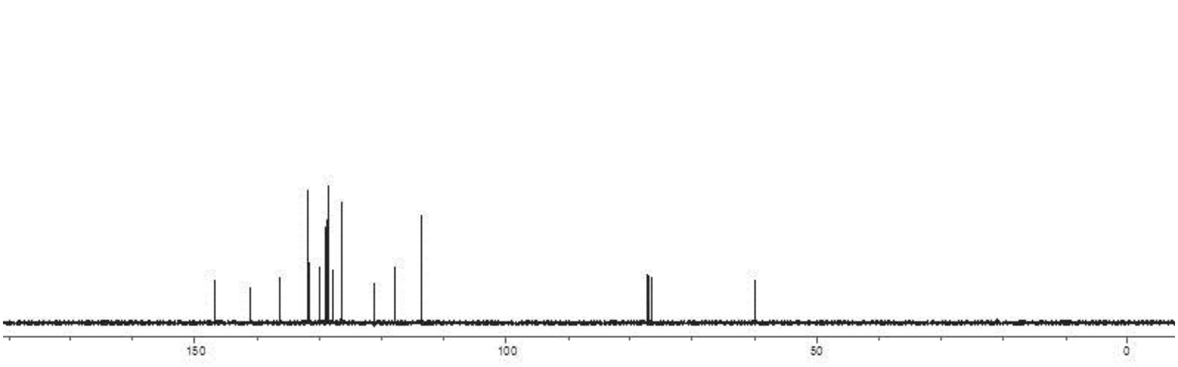

NMR spectra of 5m

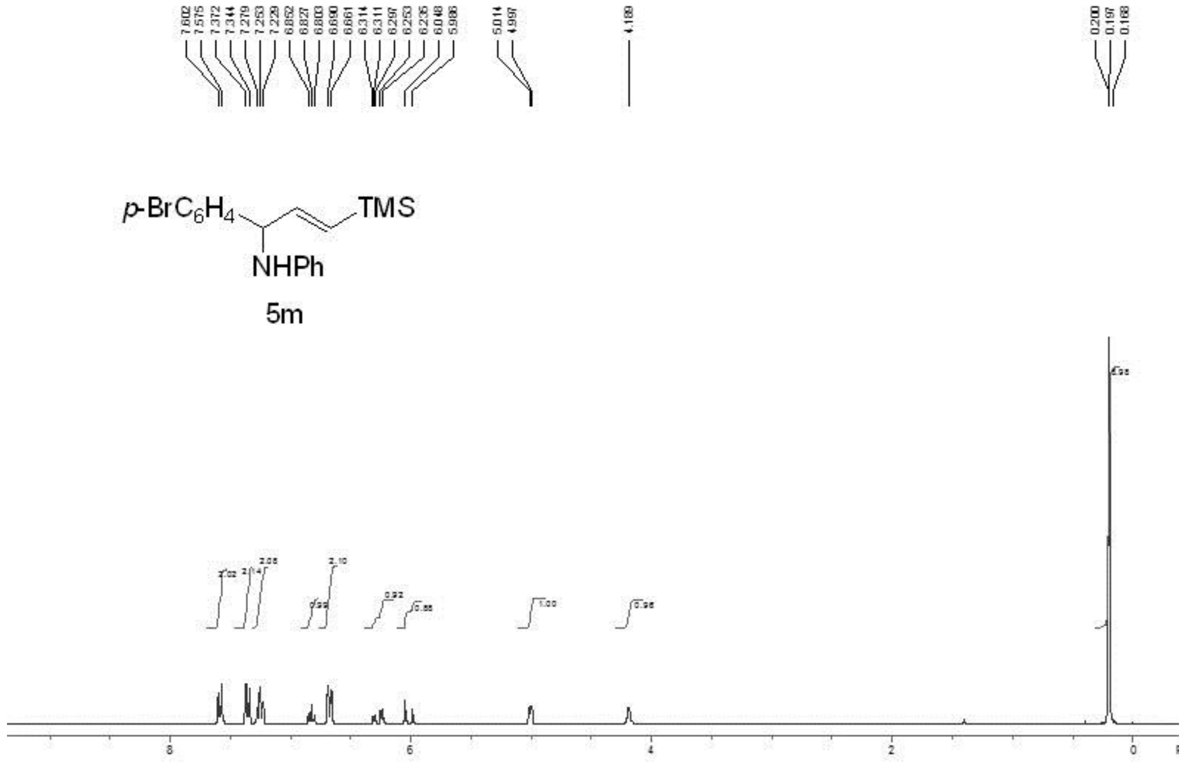

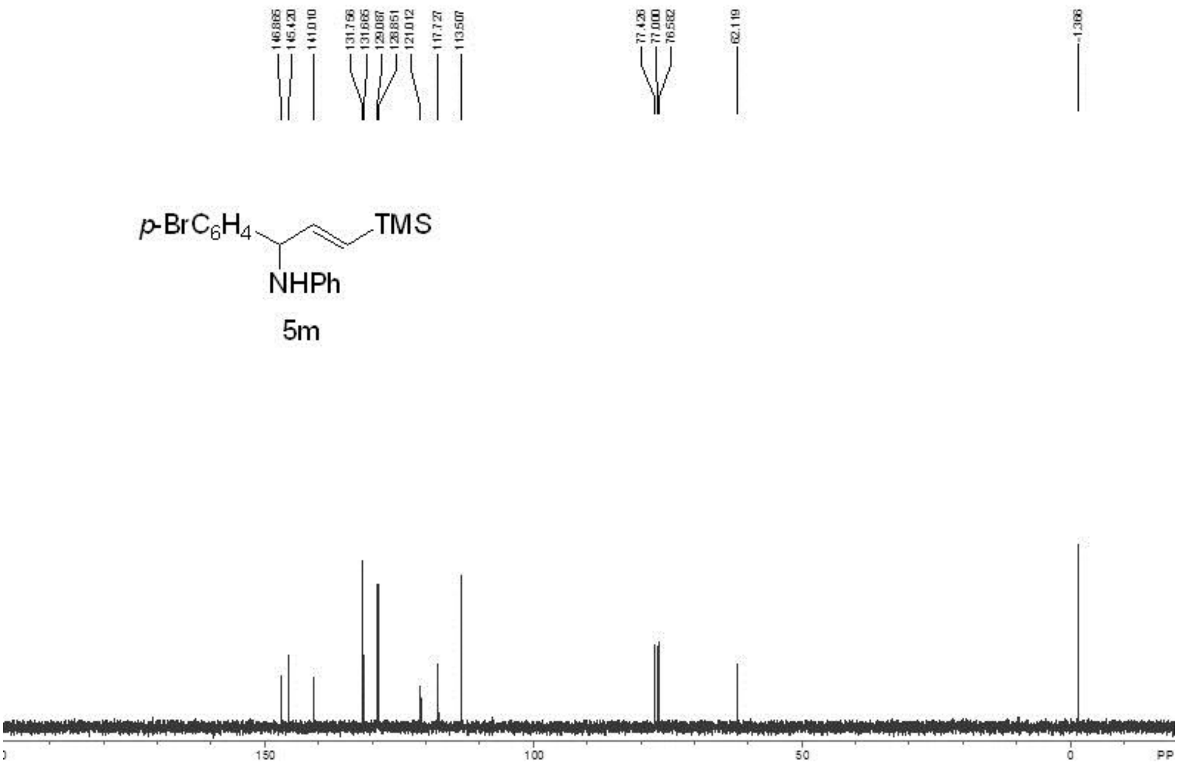

NMR spectra of 5n

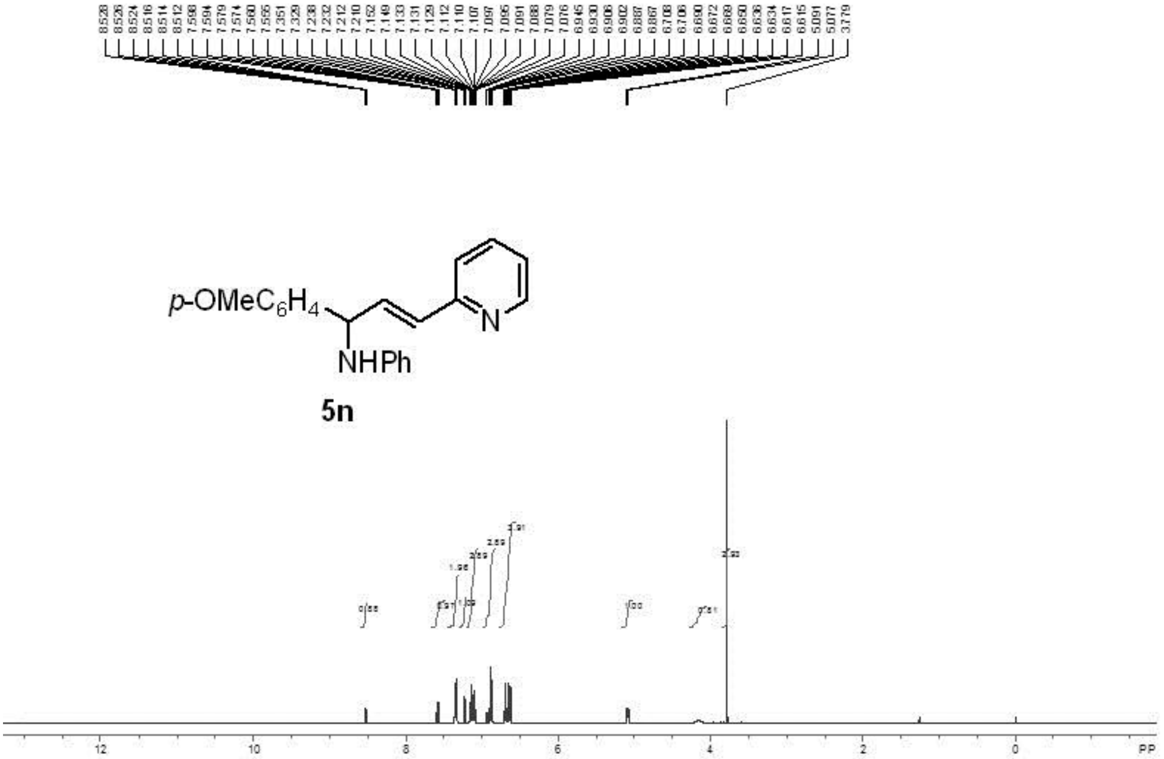

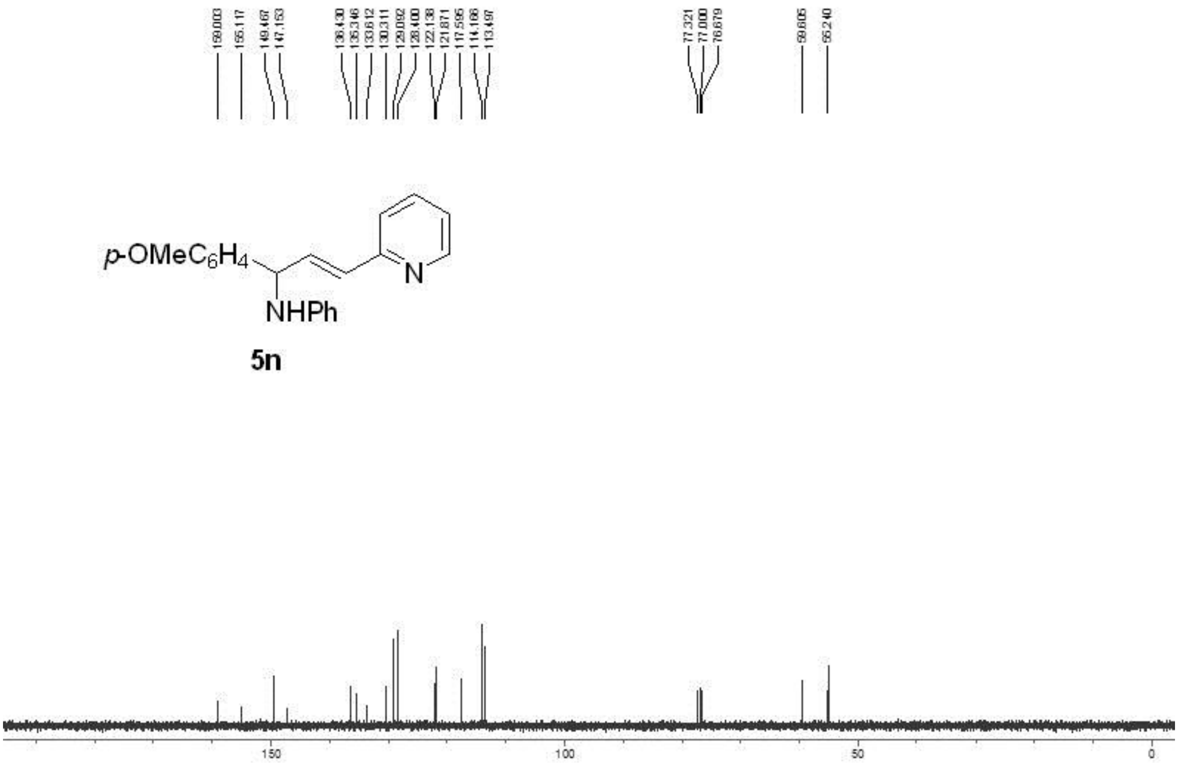

NMR spectra of 5o

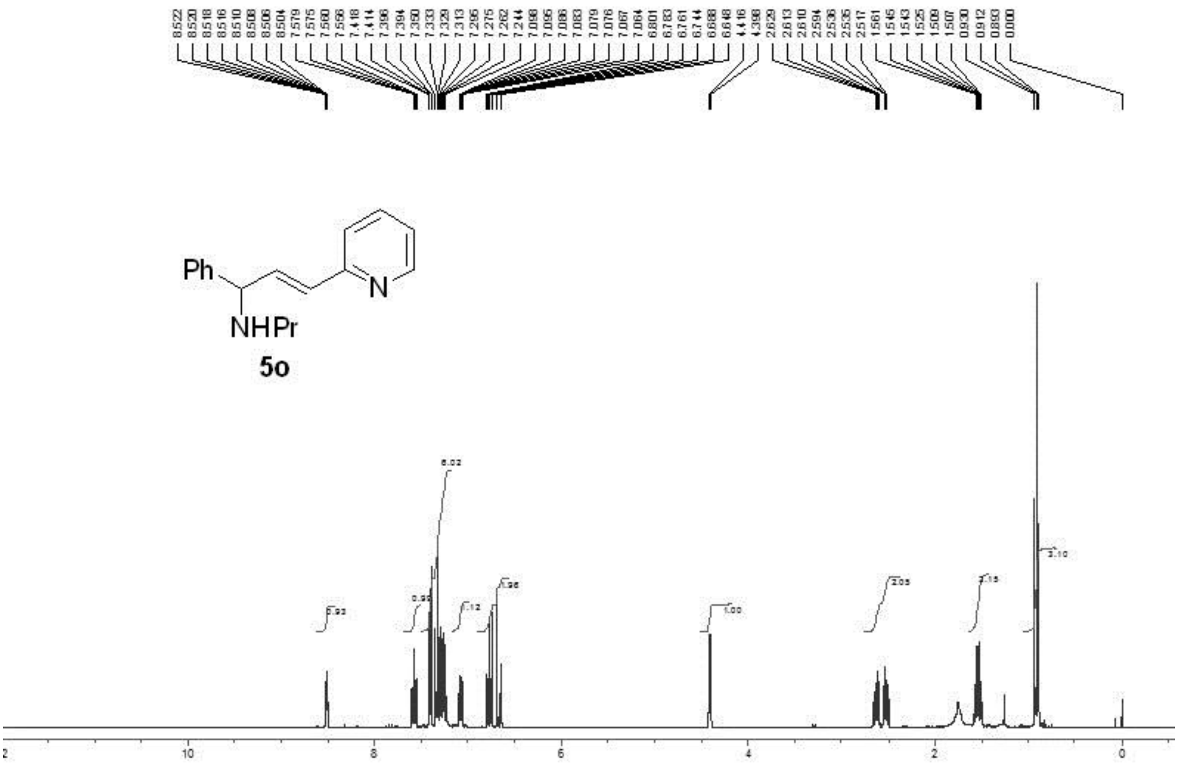

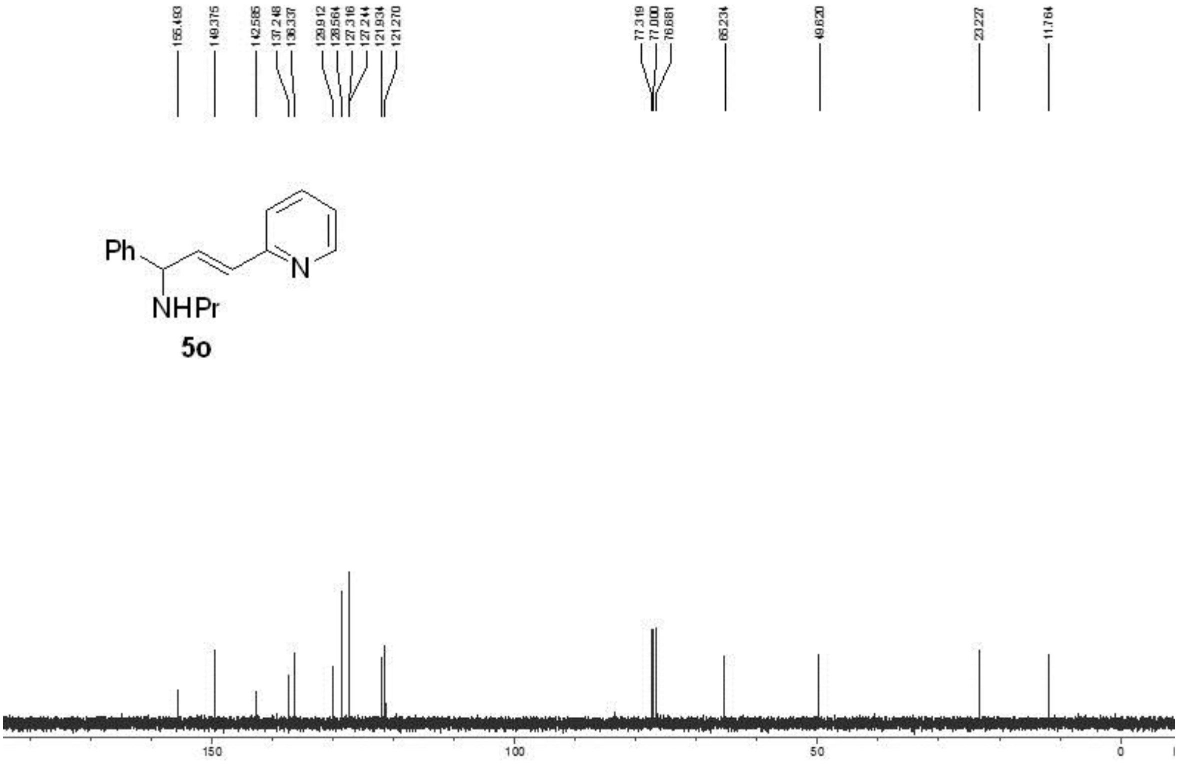

NMR spectra of 5q

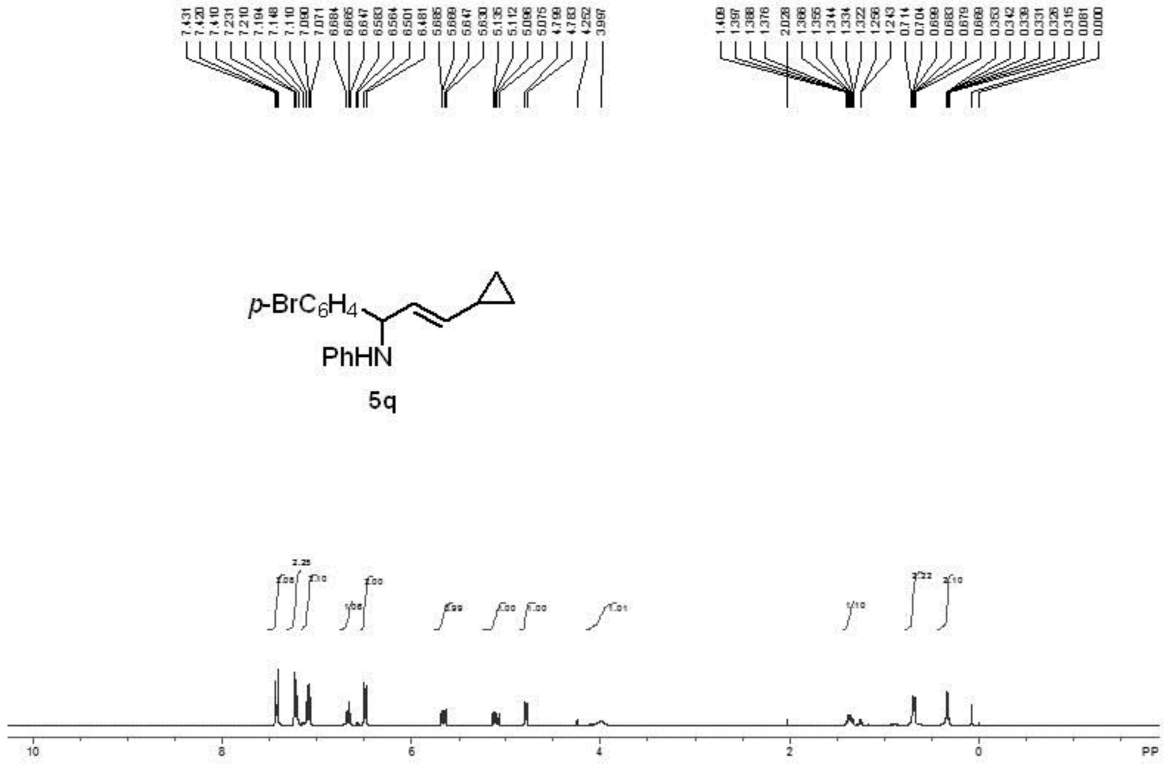

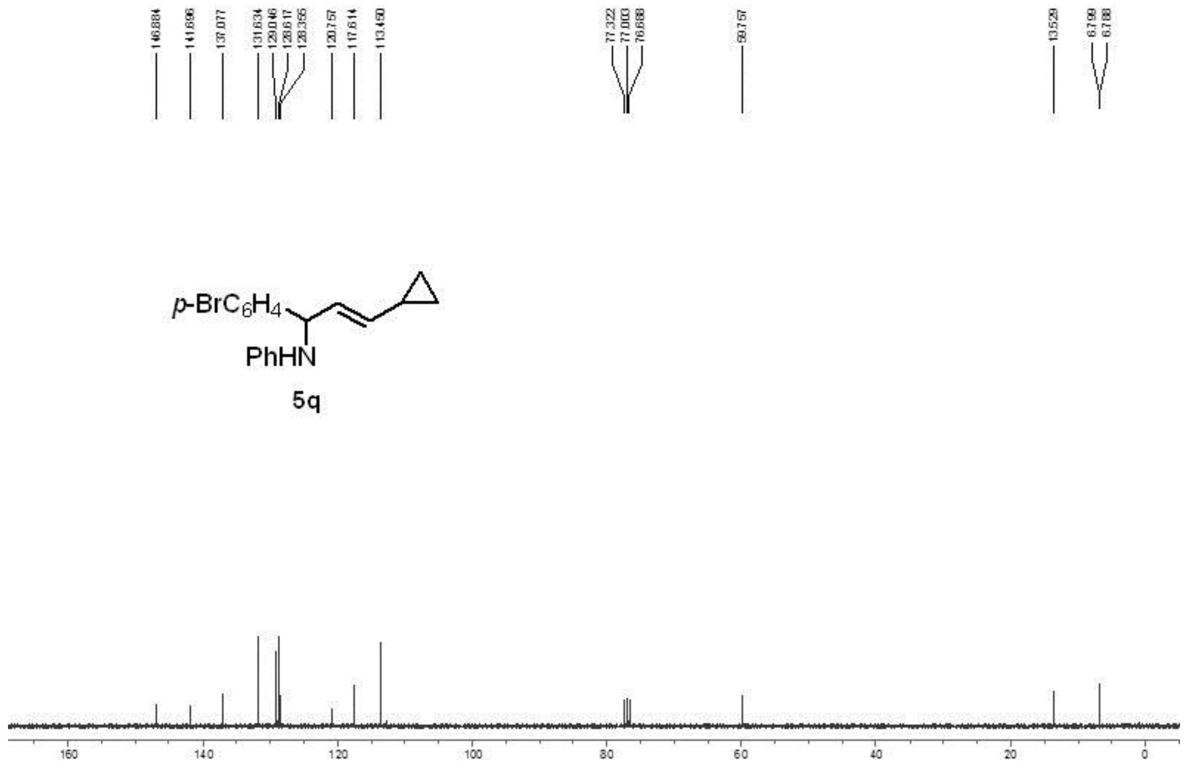

NMR spectra of 6q

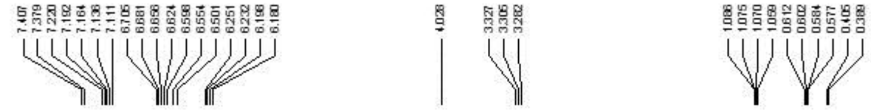

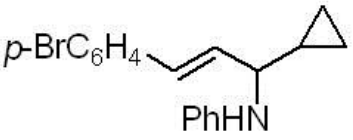

6q

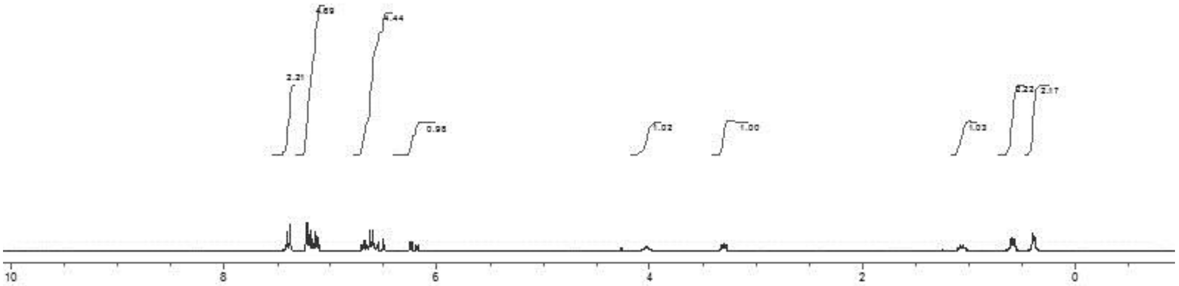

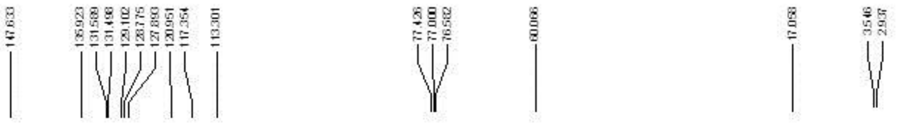

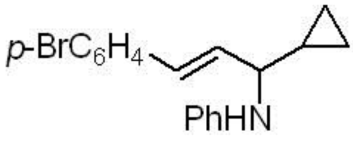

6q

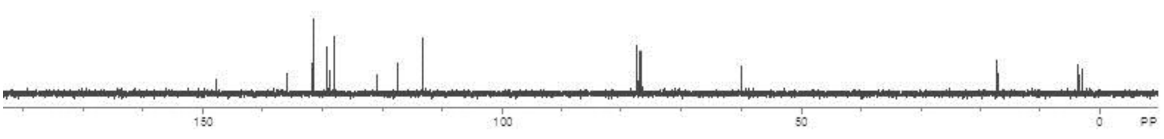

NMR spectra of 7

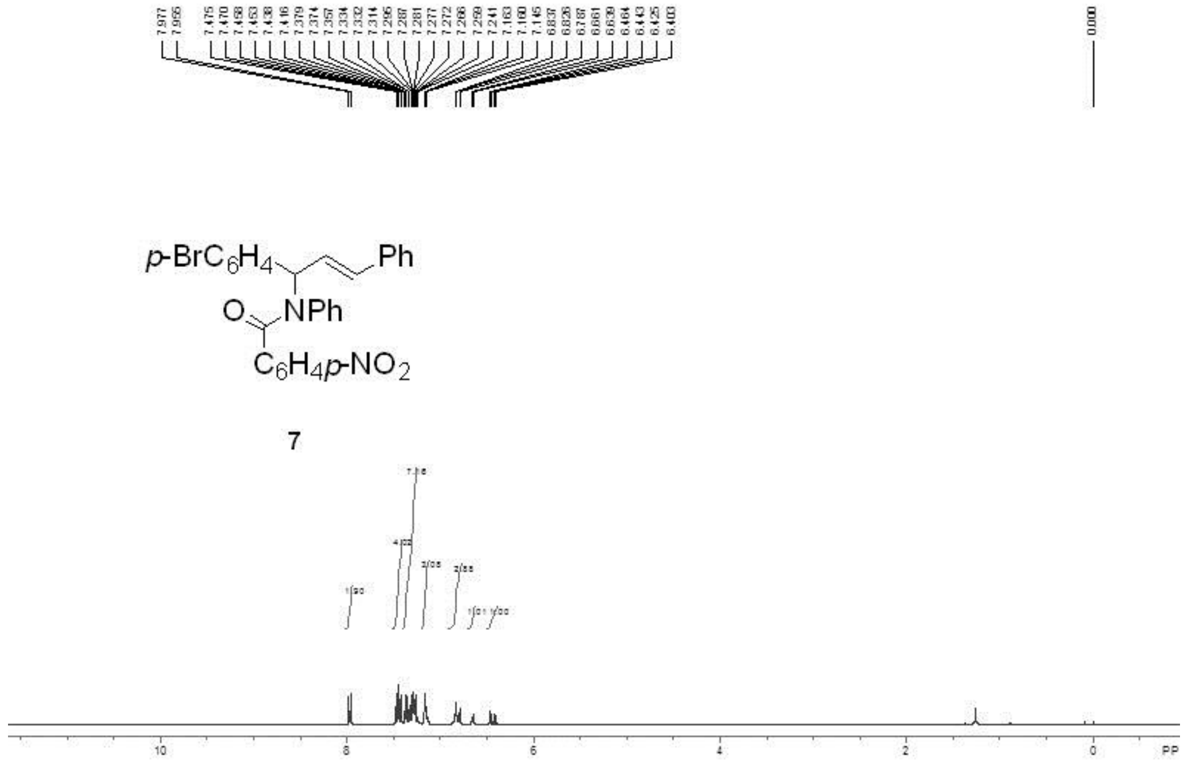

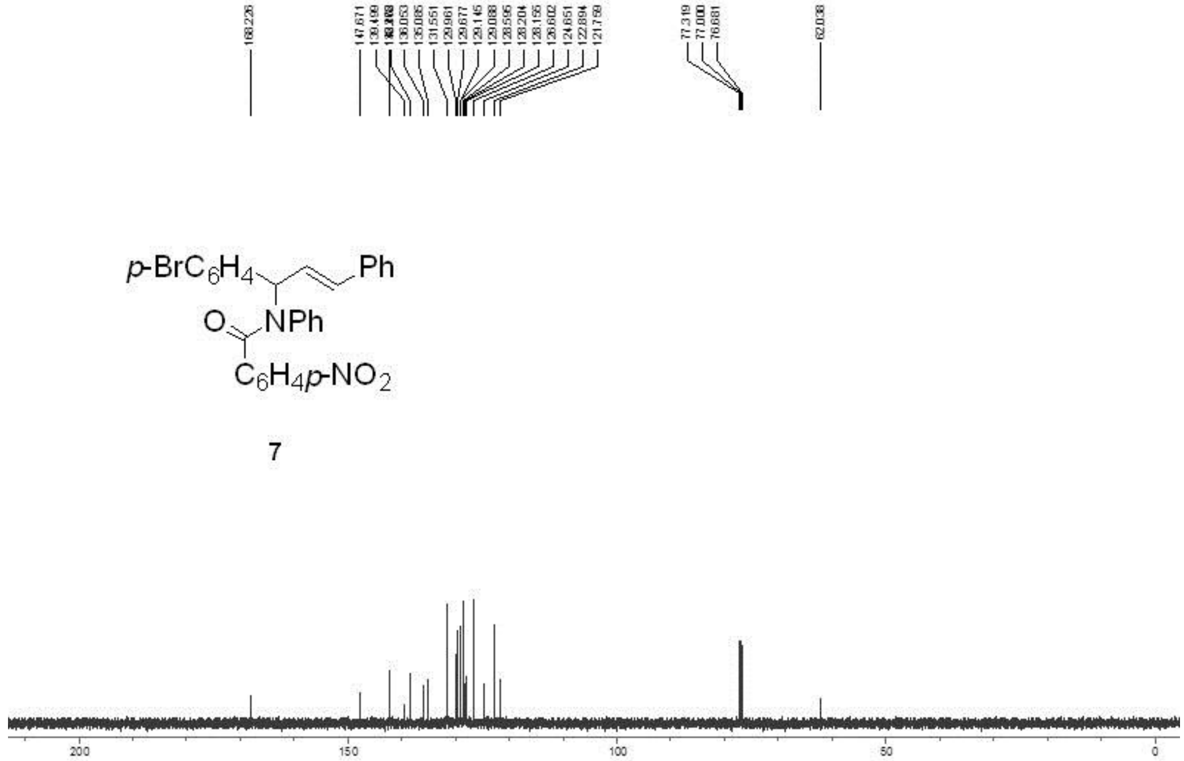

NMR spectra of 8

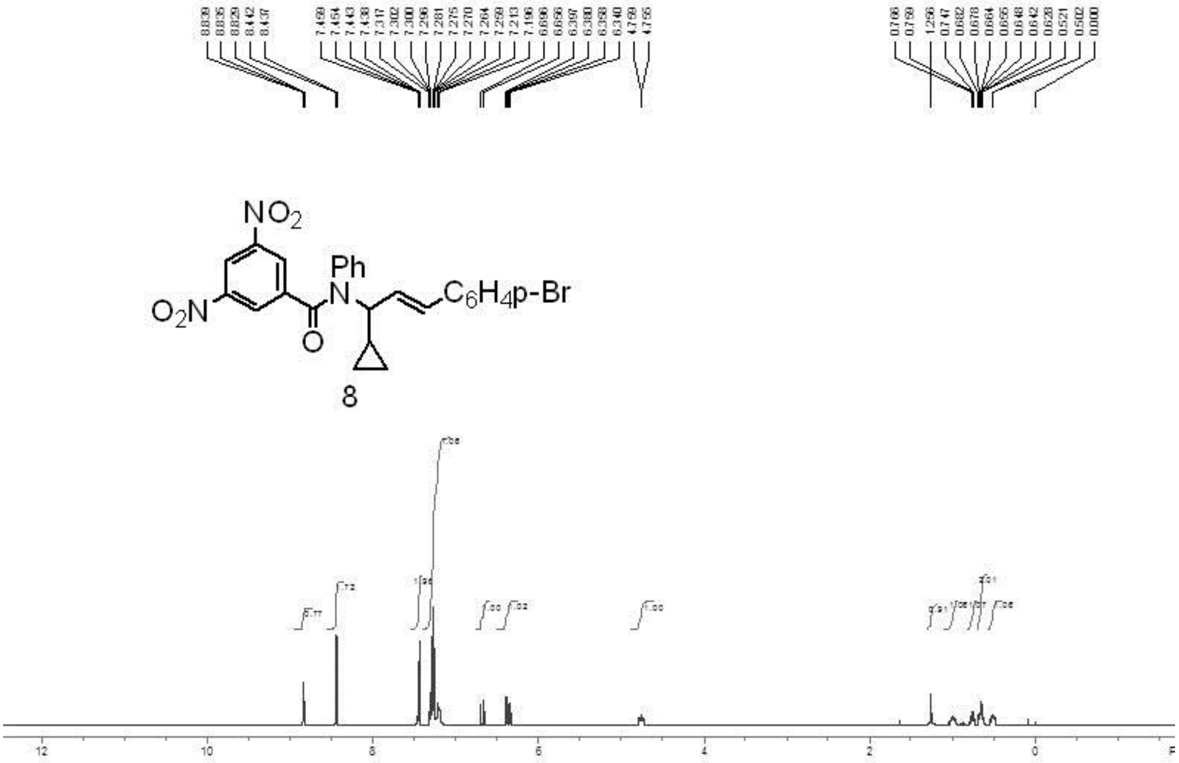

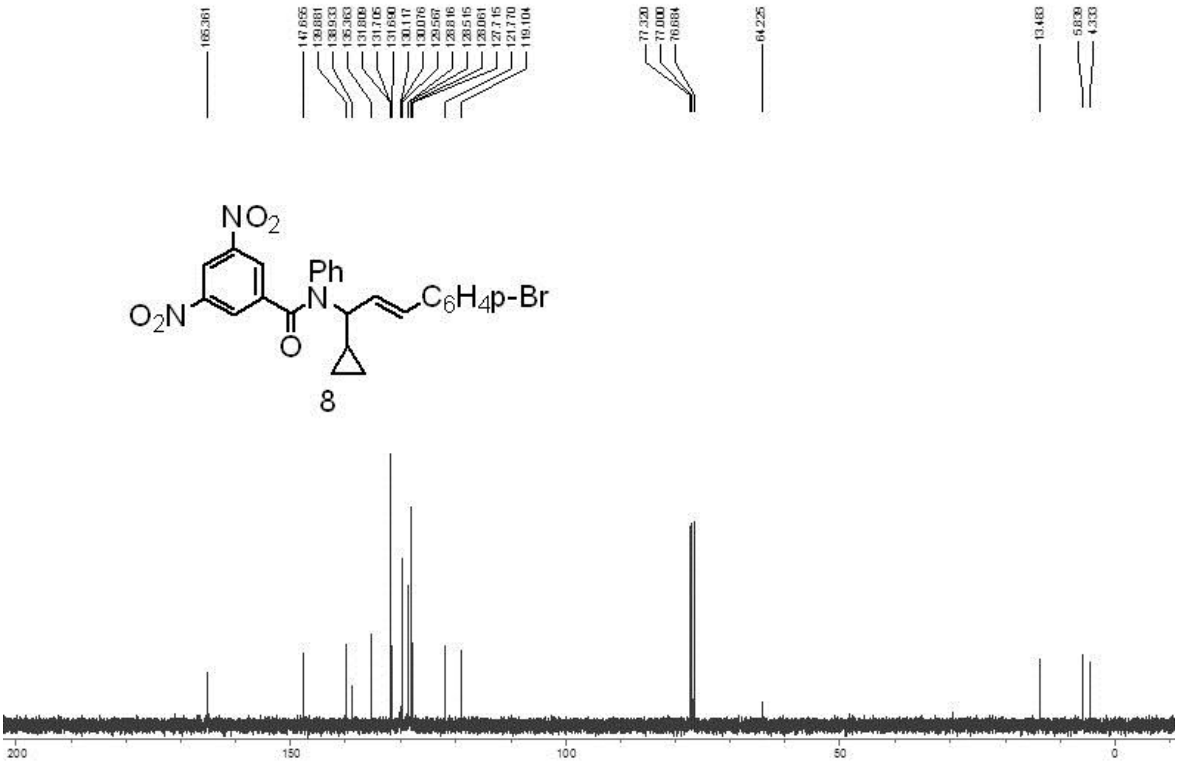

NMR spectra of 9

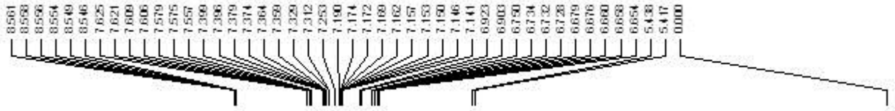

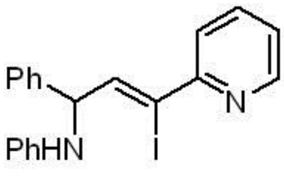

9

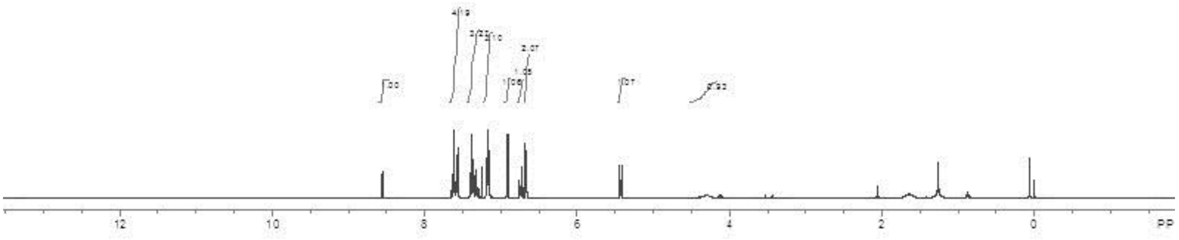

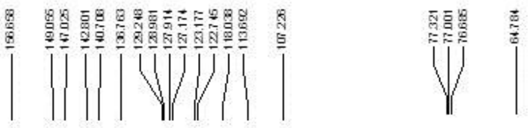

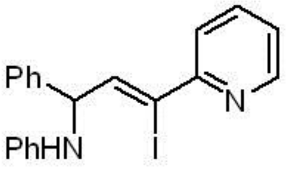

9

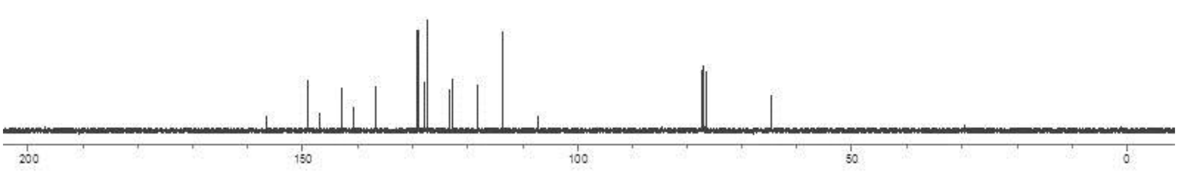

Supplement: File 1 — Experimental section and NMR spectra. [file Beilstein_J_Org_Chem-09-621-s001.pdf]
